# Post-Mastectomy Chest Wall Irradiation Effects on 10-Year Survival in Early Breast Cancer

**DOI:** 10.1056/NEJMoa2412225

**Published:** 2025-11-06

**Authors:** Ian H. Kunkler, Nicola S. Russell, Niall Anderson, Richard Sainsbury, J. Michael Dixon, David Cameron, Juliette Loncaster, Matthew Hatton, Helen Westenberg, Jackie Clarke, Heather McCarty, Rhun Evans, Konstantinos Geropantas, Virginia Wolstenholme, Abdulla Alhasso, Pamela Woodings, Lisa Barraclough, Neil Bayman, Richard Welch, Fidelis Muturi, Tracy McEleney, Jaqueline Burns, Katherine Riddle, Eve Macdonald, Johanna Dunlop, Nicole Sergenson, Geertjan van Tienhoven, Karen J. Taylor, John M.S. Bartlett, Tammy Piper, Galina Velikova, Edwin Aird, Boon Chua, Coen Hurkmans, Karen Venables, Linda J. Williams, Jeremy S. Thomas, Andrew M. Hanby, Marjory Maclennan, Susan Cleator, Eldo T. Verghese, Yexiong Li, Shulian Wang, Peter Canney

**Affiliations:** 1 Institute of Genetics and Cancer, University of Edinburgh, UK; 2 Dept. Radiation Oncology, Netherlands Cancer Institute, Amsterdam, Netherlands; 3 University of Edinburgh, Department of Public Health, UK; 4 University College London, UK; 5 Edinburgh Breast Unit, University of Edinburgh Western General Hospital, UK; 6 Clinical Oncology, The Christie NHS Foundation Trust, Manchester, UK; 7 Clinical Oncology Sciences, Weston Park Hospital, Sheffield, UK; 8 Radiotherapie Groep, Arnhem, Netherlands; 9 Belfast City Hospital, Northern Ireland, UK; 10 Norfolk and Norwich University Hospital NHS Trust, UK; 11 Barts Health NHS Trust, London, UK; 12 Beatson West of Scotland Cancer Centre, Glasgow, UK; 13 Department of Oncology, Royal Derby Hospital, UK; 14 Royal Bolton Hospital NHS Foundation Trust, UK; 15 Public Health Scotland, UK; 16 Scottish Clinical Trials Research Unit, Edinburgh, UK; 17 University of Aberdeen, UK; 18 Radiation Oncology, Amsterdam UMC Netherlands; 19 St James’ University Hospital, Leeds, UK; 20 Mount Vernon Hospital, Northwood, UK; 21 University of New South Wales, Sydney, Australia; 22 Dept. Radiation Oncology Catharina Ziekenhuis, Eindhoven, Netherlands; 23 Usher Institute of Population Sciences and Informatics, University of Edinburgh, UK; 24 Department of Pathology, Western General Hospital, Edinburgh; 25 Imperial College Healthcare NHS Foundation Trust, London, UK; 26 Chinese Academy of Medical Sciences, Beijing, China

## Abstract

**Background:**

The role of post-mastectomy chest wall irradiation in pN1 breast cancer patients with 1-3 axillary nodes or pN0 with additional risk factors is uncertain.

**Methods:**

We undertook a randomized phase 3 international trial investigating the omission of chest wall irradiation in women with “intermediate-risk” breast cancer (pT1-2N1; pT3N0 or pT2N0 with grade III and/or lympho-vascular invasion) treated by mastectomy, an axillary procedure and systemic therapy. Patients were randomized to 40 - 50 Gy chest wall irradiation (CWI) or no CWI. Primary endpoint was overall survival, with 10 years of follow-up. Chest wall and regional recurrence, disease-free survival, distant metastasis-free survival, causes of death and morbidity were also assessed.

**Results:**

In the intent to treat population, 808 patients were randomized to CWI and 799 to no CWI. Median follow up was 9.6 years. There was no evidence of a difference in overall survival, 81.4% with CWI, 81.9% without CWI (10-year Kaplan-Meier estimates), hazard ratio (HR) of 1.04; 95% confidence interval [CI], 0.82 to 1.30, (p=0.80). There were 29 chest wall recurrences (9 with CWI, 20 without CWI), representing <2% absolute difference; HR 0.45; 95% CI, 0.20 to 0.99. Disease-free survival was 78.2% with CWI and 75.5% without CWI; HR 0.97; 95% CI, 0.79 to 1.18, and distant metastasis-free survival was 78.2% with CWI and 79.2% without CWI; HR 1.06; 95% CI, 0.86 to 1.31.

**Conclusion:**

In this phase 3 trial of patients with intermediate-risk early breast cancer treated by mastectomy and contemporary adjuvant systemic therapy, chest wall irradiation did not improve overall survival. (Funded by UK MRC, and others; Trial Reg:)

## Introduction

Mastectomy is standard of care for over one third of patients with stage I and II breast cancer.^(1)^ Stage II disease includes tumors ≤ 5 cm with one to three axillary lymph node metastases (N1), or tumors ≥ 2cm without nodal metastases (TNM stages T1-2N1M0 and T2-3N0M0). Stage II patients with either N1 disease or N0 but with poor histological features (including larger size, grade III histology or lymphovascular-invasion [LVI]) are considered at “intermediate-risk” of recurrence.^(2)^ Landmark Danish and Canadian randomized controlled trials (RCTs) reported in 1997-99 showed that post-mastectomy radiotherapy for stages II and III reduced the risk of locoregional recurrence and improved 10-year survival in women with nodal metastases.^(3–5)^ The 2014 EBCTCG meta-analysis of trials of post-mastectomy radiotherapy,^(6)^ which relies heavily on the Danish and Canadian trials, showed a 16.5% reduction in ‘locoregional recurrence first’ and a 7.9% gain in 20-year survival in patients with pN1 disease. Adjuvant systemic therapy in these trials is now considered sub-optimal.^(7, 8)^ Subsequently, major improvements in systemic therapy and reductions in breast cancer mortality^(9)^ challenge the applicability of the evidence base for post-mastectomy radiotherapy to current practice. Hence, the role of post-mastectomy radiotherapy in patients with 1-3 involved axillary nodes is uncertain as reflected in differing guidelines^(10–12)^ and practice.^(13, 14)^ Post-mastectomy radiotherapy in patients with 1-3 involved nodes became a research priority of the NIH in 2000.^(15)^ Intermediate-risk pN0 patients might also benefit from post-mastectomy radiotherapy.^(16)^ Most locoregional recurrences occur on the chest wall,^(17, 18)^ so this is considered a critical target for post-mastectomy radiotherapy. We present a more contemporary picture of the impact of post-mastectomy radiotherapy selectively to the chest wall on overall survival in the 10-year results of the BIG 2.04 MRC/EORTC SUPREMO trial.

## Methods

### Oversight

The BIG 2-04 MRC/EORTC SUPREMO (Selective Use of Postoperative Radiotherapy after Mastectomy) is a multicenter, phase 3 randomized clinical trial.^(19)^ The trial recruited at 125 UK sites, 27 sites in 9 European countries and an additional 21 international sites (table S1.1, available at NEJM.org). The protocol received UK ethical approval (MREC Ref: 05/S0501/106) and equivalent approval in non-UK jurisdictions. All patients provided written informed consent. UK patients could consent to participation in the Quality of Life (QoL), Cardiac and Health Economics substudies. UK and EORTC patients could participate in the translational TRANS-SUPREMO substudy. ISRCTN registration is 61145589. The Trial Management Group (including patient representation) designed the trial (S1.2). The Scottish Clinical Trial Research Unit in Edinburgh provided the data management and the remote monitoring to ensure adherence with Good Clinical Practice. The Trial Steering Committee and Data Monitoring and Ethics Committee provided trial oversight (S1.3.and S1.4). NA performed the analysis.

IK wrote the first manuscript draft. The authors wrote the article and vouch for the accuracy and completeness of the data and for trial protocol compliance. The funders had no role in the design, analysis or publication of the data.

### Patient eligibility criteria

Eligible women had undergone a mastectomy for unilateral stage II intermediate-risk breast cancer without distant metastases (specified as pT1-2N1; pT2N0 if also grade III and/ or LVI) and had a minimum clear margin of 1 mm, including patients undergoing immediate breast reconstruction.

Following the amended v29 protocol (2010), patients were also eligible with stage II breast cancer, with T3N0 and after neo-adjuvant chemotherapy.

A level II axillary nodal clearance of a minimum of 10 nodes (protocol v.27) or 8 nodes (protocol v.29) was mandatory for N1 patients. For pN0 patients, axillary staging was by four-node axillary node sample, a sentinel node biopsy or axillary node clearance. Full inclusion and exclusion criteria of both protocols are given in the supplement S2.1.

### Treatment and trial procedures

Patients were randomized 1:1 by permuted blocks with block length varied to minimize the effects of entry bias, with stratification by treating center.

The protocol specified guidelines for pathology, surgery, radiation and adjuvant systemic therapy. We reviewed the pathology reports of all patients in the trial to check eligibility (S 2.2). Two pathologists (JT, AH) conducted a central pathology review of histology slides from UK and EORTC patients.^(20)^

Patients randomized to CWI received doses ranging from 40 Gy in 15 fractions to 50 Gy in 25 fractions. The clinical target volume encompassed the chest wall skin flaps and soft tissues from 5 mm under the skin surface down to the deep fascia. Axillary radiation was not permitted, but centers could elect to irradiate the supraclavicular fossa or internal mammary chain for N1 patients (UK only) or N0/N1 for non-UK sites, irrespective of allocation of CWI. Radiotherapy was quality assured at institute and patient level. For the full radiotherapy and RT-QA protocols, see S2.3 at NEJM.org.

Adjuvant or neoadjuvant chemotherapy with anthracyline-containing regimens ± taxanes was recommended. Trastuzumab was administered according to local policy. Patients with ER positive tumors were recommended a minimum of 5 years of adjuvant endocrine therapy.

Tamoxifen/aromatase inhibitor or a sequential combination was advised for postmenopausal patients, and for premenopausal women tamoxifen, ovarian ablation or both.

Patients attended follow-up clinic appointments 3 months post treatment in the first year, thereafter annually from the date of mastectomy for 10 years. Contralateral mammography, if appropriate, was recommended biannually for 10 years. Follow-up and toxicity forms were completed at each visit. Toxicity, using the EORTC/RTOG Radiation Morbidity Scale, was assessed annually for 10 years.^(21)^ Acute morbidity was assessed at the end of radiotherapy, or for non-irradiated patients at 3 months after surgery if they had not received chemotherapy, or at 3 months after chemotherapy.

### Trial endpoints

The primary outcome was overall survival. Secondary outcomes were chest wall recurrence (± recurrence elsewhere); regional recurrence; disease-free survival; distant metastasis-free survival; cause of death; acute and late radiation morbidity; quality of life and cost effectiveness. Quality of life and cost-effectiveness analyses will be reported separately.

### Statistical analysis

The null hypothesis was that there is no effect of CWI on overall survival in women with intermediate-risk breast cancer treated by mastectomy, axillary surgery and adjuvant systemic therapy.

Protocol (v.27) specified a target sample size of 3500 patients to have 80% power to detect a significant difference at the 5% level under a superiority design when the five-year survival rates ± CWI were 75% and 79% with accrual over 4 years. As initial accrual was slower than projected, we reduced the sample size, extended the follow-up to a 10-year period and widened the eligibility criteria including neoadjuvant systemic therapy (S2.1). The extension of follow-up to 10 years was supported by a subgroup analysis of node positive patients from the Danish trials that showed a 9% survival advantage to the irradiated group at 15 years (57% vs 48%) (p=0.003). This survival advantage only emerged after 5 years.^(22)^

Revised sample size and powering were based on an assumption of a difference of 7% in overall survival (56% without CWI vs 63% with CWI) between the arms of the trial (corresponding to a hazard ratio of 1.255) with 80% power at the 0.05 level of significance. The sample size to detect this difference would be 1600 patients allowing for 5% loss to follow up (or 609 events). An ethically and funder approved modification to the protocol, v.29, was made. All analyses were based on the intention-to-treat (ITT) principle, and two-tailed significance tests and confidence intervals used throughout. No multiplicity adjustments were made – see S4.3. Analysis of overall survival and other time-to-event outcomes was based on the calculation of 95% confidence intervals for the hazard ratios from a Cox proportional hazards model, adjusting for three geographical clusters of center (UK, Europe, other international). Proportional hazards checks were made for every covariate by Schoenfeld residuals score tests (S4.3). Kaplan-Meier calculations and plots were used for graphical display and estimation of rates of endpoints at 10 years. We report three of the most relevant pre-specified subgroup analyses– age-group, nodal status, molecular subtype, performed by estimation of strata-specific estimates and confidence intervals.

## Results

**Figure 1:**
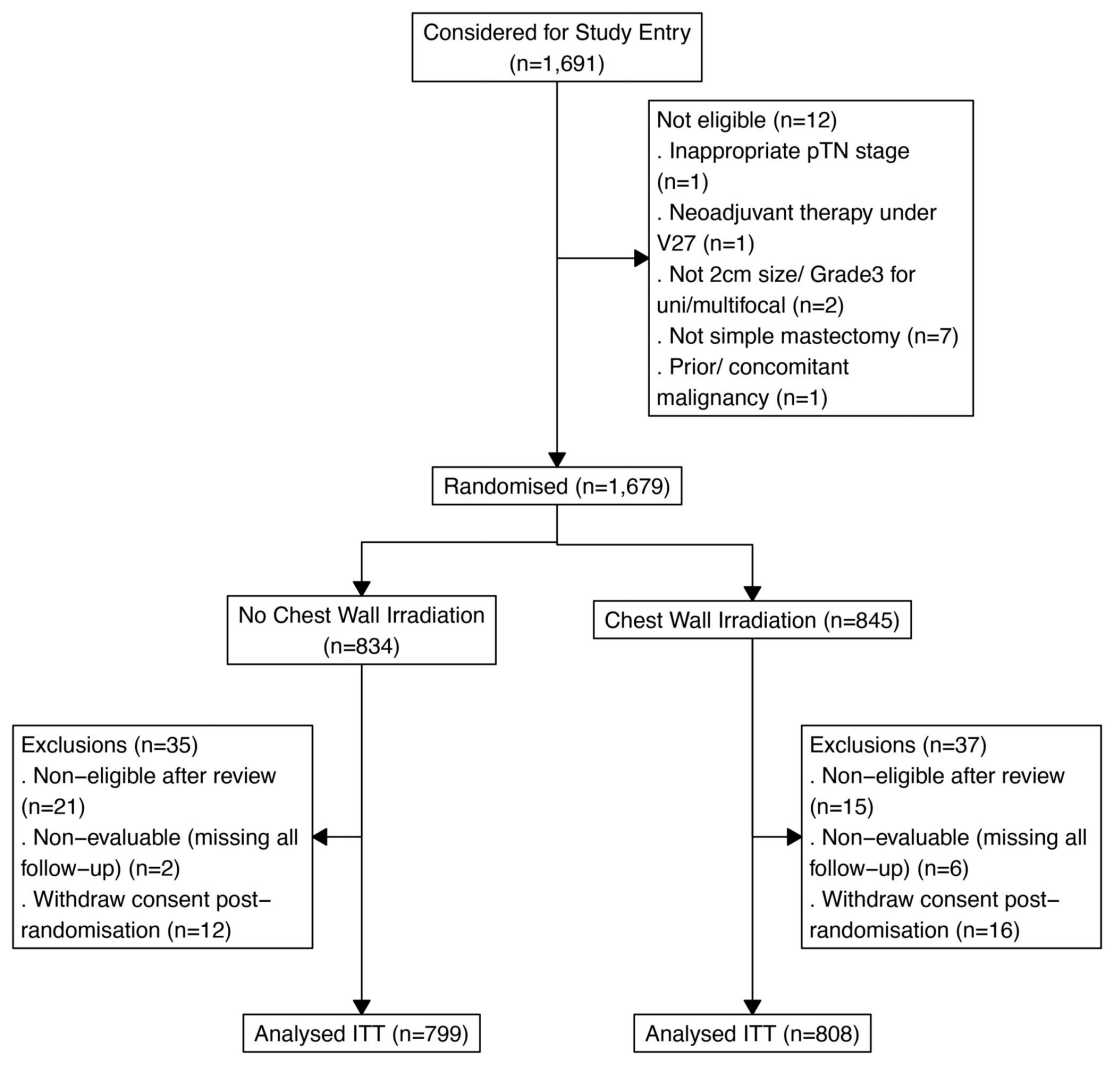
CONSORT Diagram

The consort diagram and patient characteristics in the ITT population are shown in Fig. 1 and Table 1. Additional tumor and treatment details are provided in table S3.1. In the ITT population, 808 patients were randomized to CWI and 799 to no CWI (August 4, 2006 - April 29, 2013). Supraclavicular fossa was irradiated in 97/808 patients with CWI and 12/799 patients without CWI. Internal mammary chain was irradiated in 12/808 patients with CWI, and 7/799 patients without CWI. In total 85% of patients received chemotherapy (87% delivered per local protocol), 79% endocrine therapy and 19% trastuzumab. Median follow up was 9.6 years. The trial database was locked on June 19, 2024.

The QoL sub-study included 78% of the UK patients.^(19)^ TRANS-SUPREMO collected tumor tissue and blood from 1397 (93.7%) UK and EORTC patients.

### Primary endpoint

**Figure 2:**
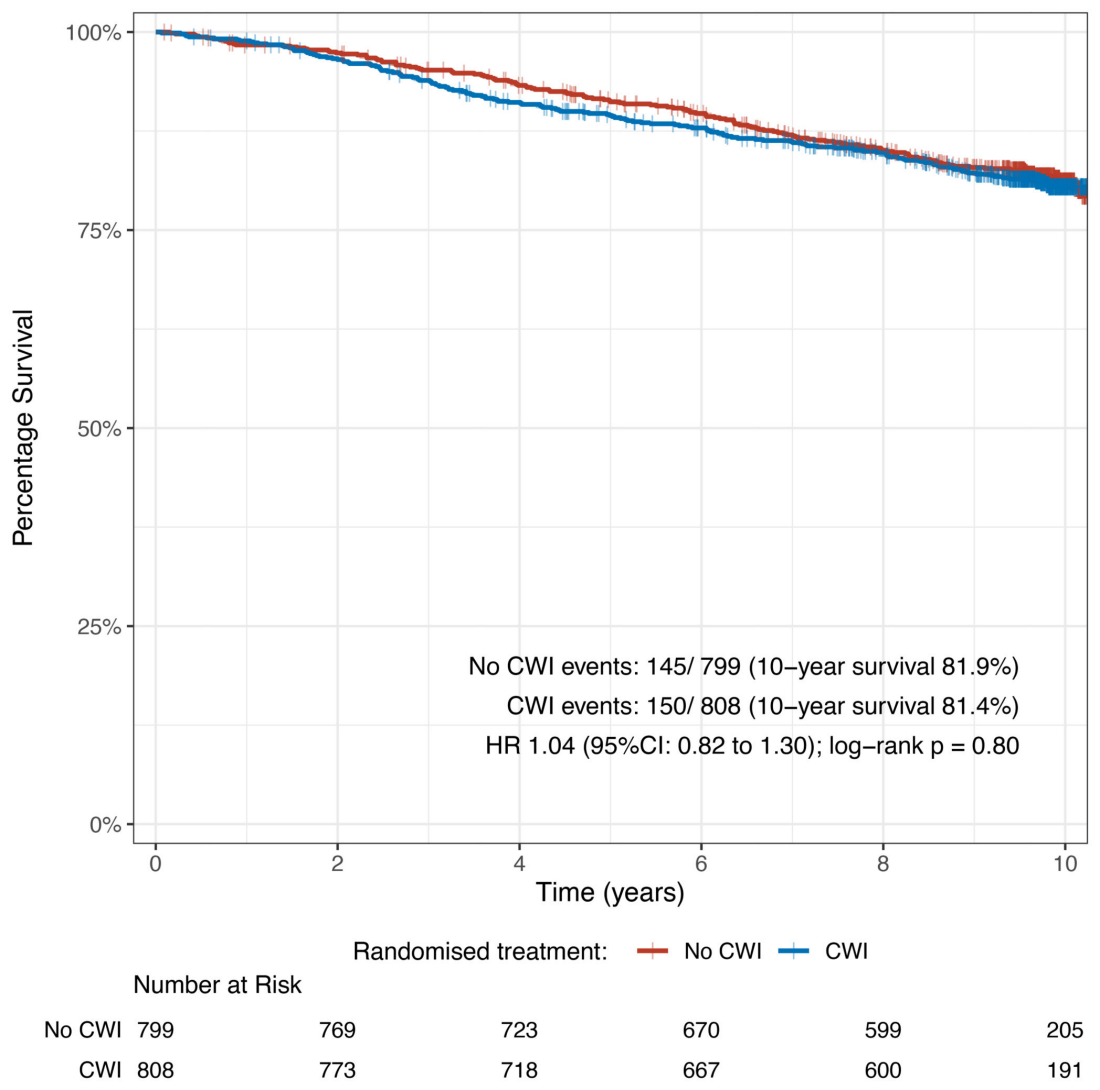
Kaplan-Meier plot for unadjusted overall survival in ITT population. Note that for clarity, the horizontal scale has been truncated at 10 years (excluding a small proportion of follow-up extending beyond this time period).

We observed 295 primary endpoint events (150 in the irradiation group and 145 in the no irradiation group). There was no evidence of a difference in overall survival with chest wall irradiation: estimated at 10 years as 81.4% with irradiation, 81.9% without irradiation (Fig. 2); hazard ratio [HR] 1.04, 95% confidence interval [CI], 0.82 to 1.30, p=0.80. Most deaths (195/295, 65.8%) were due to breast cancer (S3.2).

### Secondary endpoints

**Figure 3:**
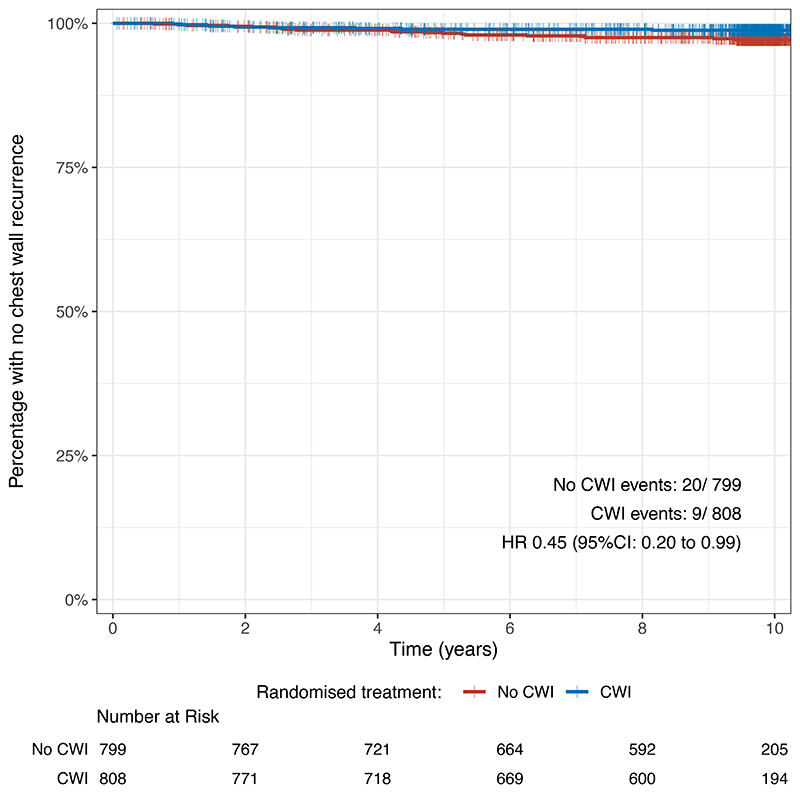

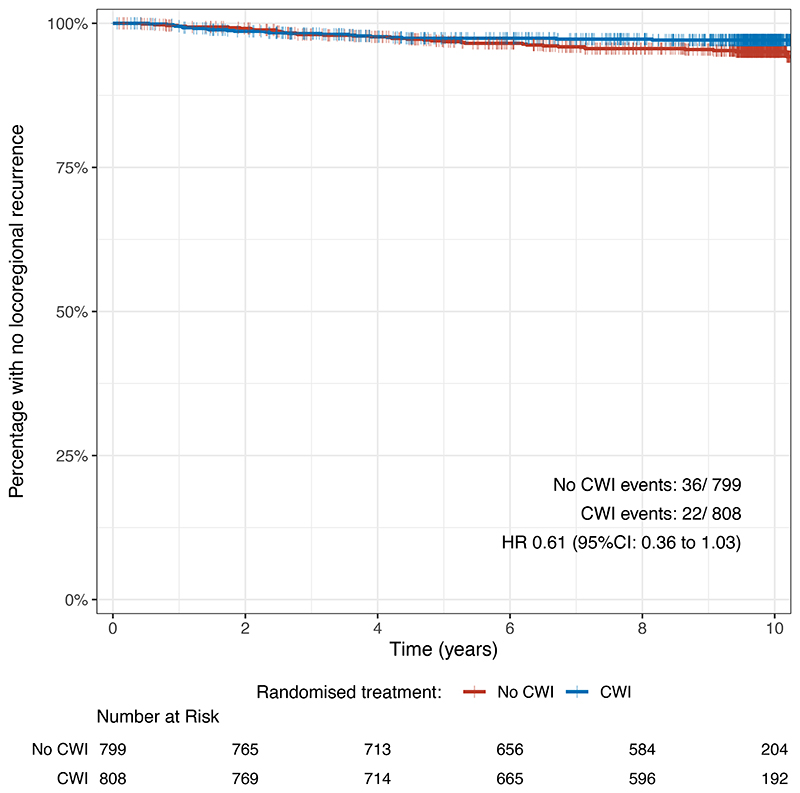

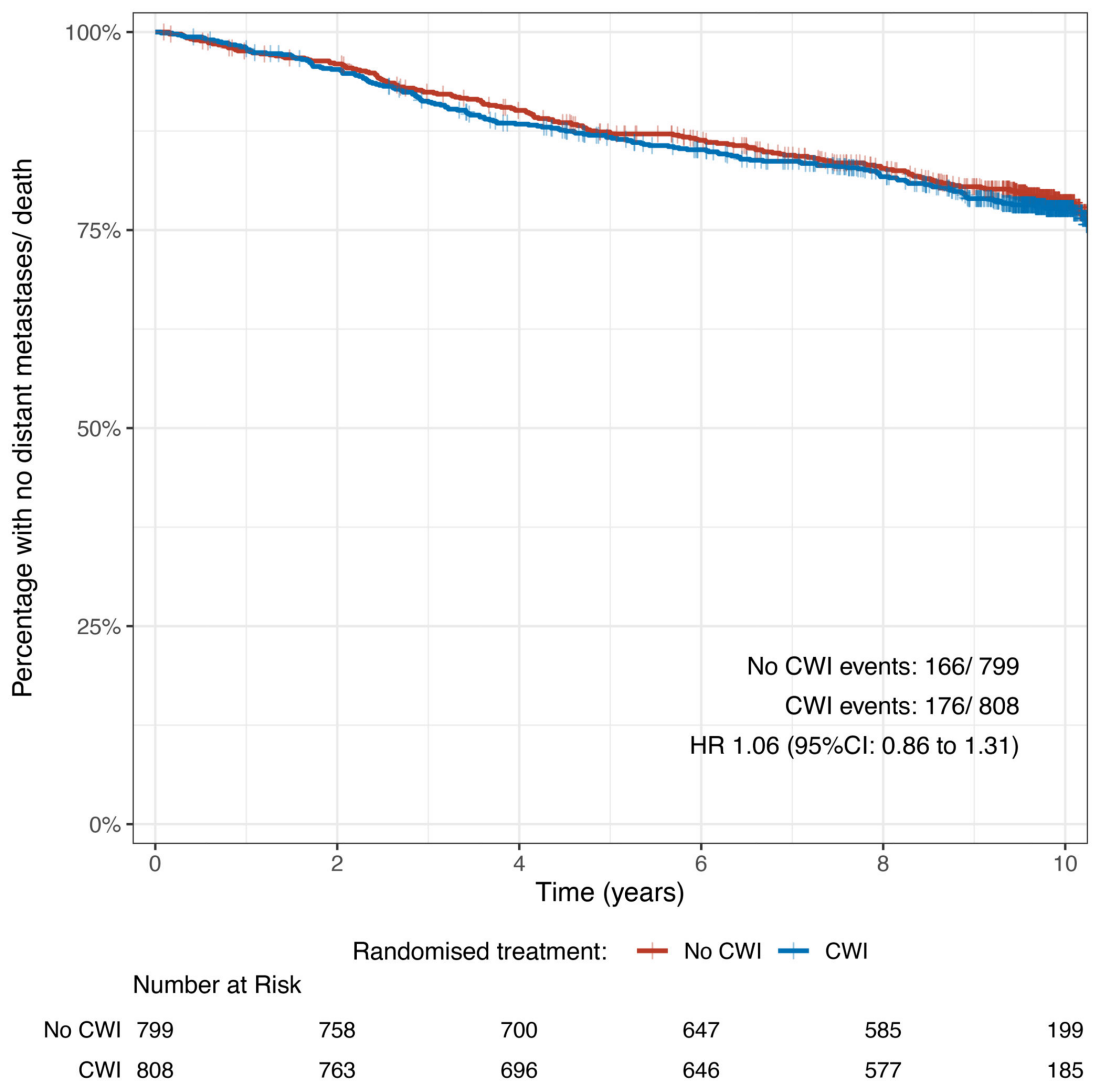

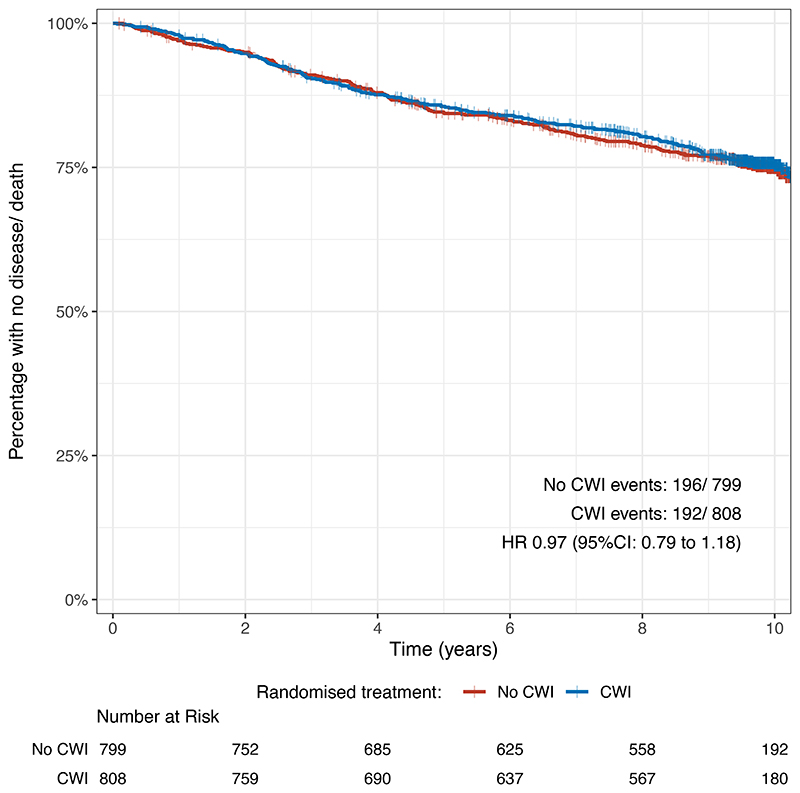
Kaplan-Meier plots for unadjusted secondary endpoints in ITT population. Note that for clarity, the horizontal scale excludes follow-up longer than 10 years. A: Chest wall recurrence-free survival. B: Locoregional recurrence-free survival. C: Distant metastasis-free survival. D: Disease-free survival.

Only 29 chest wall recurrences occurred, 20 (2.5%) without and 9 (1.1%) with irradiation, an absolute difference of <2%. A reduction in chest wall recurrence with irradiation (HR 0.45; 95% CI, 0.20 to 0.99) was seen (Fig. 3A); the confidence interval is wide due to a low number of events.

We observed 58 locoregional recurrences, 36 (4.5%) without irradiation and 22 (2.7%) with irradiation; HR 0.61; 95% CI 0.36 to 1.03, Fig 3B.

For distant metastasis-free survival we observed 176 metastatic events including death with irradiation (23.8%) and 166 events (20.8%) without irradiation; HR 1.06; 95% CI, 0.86 to 1.31, (Fig 3C).

For disease-free survival, 388 events of breast cancer recurrence or death were noted: 192 (23.8%) with irradiation, 196 (24.5%) without irradiation. Ten-year estimated disease-free survival was 76.2% with CWI; 75.5% without CWI; HR 0.97, 95% CI, 0.79 to 1.18 (Fig.3D).

### Pre-planned sub-group analyses

**Figure 4:**
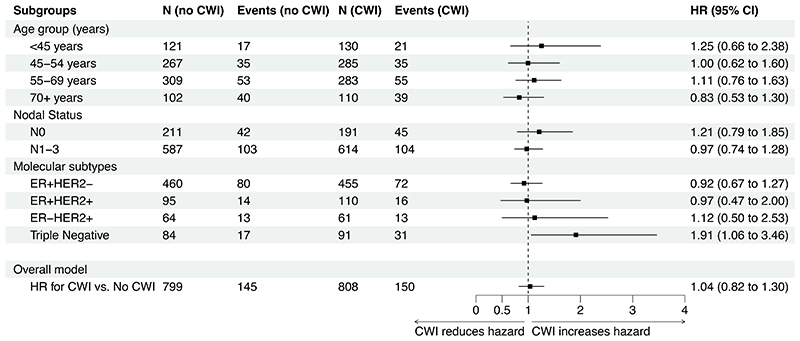

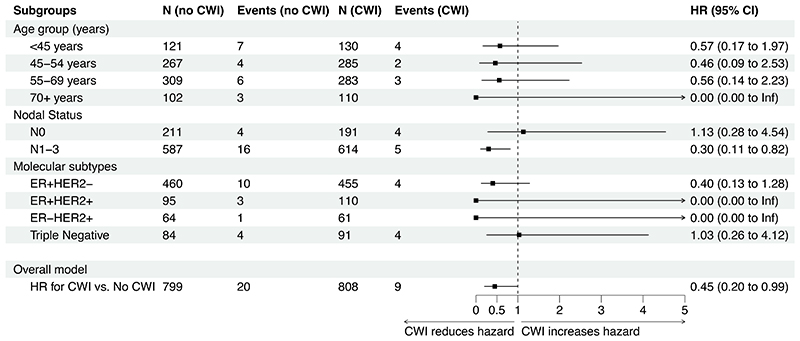
Forest plot for subgroup analyses of primary endpoint of overall survival in ITT population. Subgroups considered are age groups, nodal status and molecular subtype. For comparison, the HR for randomised treatment (stratified only by center) is provided at the bottom of the plot.

We observed no differential effect of irradiation on overall survival related to nodal status (fig 4). The number of events for pN0 patients: 191 with irradiation, 211 without irradiation; for pN1 patients: 614 with irradiation, 587 without irradiation; (HR 0.82, 95% CI, 0.63 to 1.05) for pN1 relative to pN0. Fig. S.3.4A gives the Kaplan-Meier plots for overall survival by nodal status.

We saw no differential effect of nodal status (N0 vs N1) on any of the secondary outcomes, (see forest plots and hazard ratios in fig. S3.5A-D). Figure S3.4B-D gives the Kaplan-Meier plots for local recurrence, distant metastasis-free survival, and disease-free survival by nodal status.

There was no differential effect of CWI on primary and secondary outcomes by age-group (Fig. 4 and S3.5A-D). The Kaplan-Meier plots per age-group are given in S3.6.

From the subgroup analysis by molecular subtype, we observed no differential effect of CWI on overall survival for ER+HER2-, ER+HER2+ or ER-HER2+ subtypes (fig. 4). The exception was patients with triple negative breast cancer (TNBC), who appeared to have worse overall survival with CWI (HR 2.05; 95% CI, 1.05 to 4.02), Fig. 4 and S3.7.

For chest wall recurrence, there was a paucity of events, but there seemed to be a reduced hazard of chest wall recurrence with CWI, adjusted for center and subtype; HR 0.44, 95% CI (0.19 to 1.02) but no reduction for TNBC patients, Fig. S3.5A and S3.8A-B.

Other secondary endpoints in relation to molecular subtypes are shown in S3.5B-D.

### Safety

Safety information on all 1607 patients in the ITT analysis is summarized in S3.3. Radiotoxicity was mild. Lung toxicity of grade ≥2 was <2% overall but shows the greatest difference by treatment arm (13 with CWI versus 5 without, OR = 2.59; 95% CI, 0.97 to 8.12). Heart and bone toxicity show less difference by treatment arm. Causes of death are given in S3.2. Cardiac deaths occurred in 8/799 (1%) patients in the no CWI group, and 6/808 (0.7%) in the CWI group. Lung cancer caused death in 7/799 (0.9%) patients in the no CWI group and 7/808 (0.9%) in the CWI group.

## Discussion

We show no evidence of an effect of CWI in intermediate-risk breast cancer on overall survival, disease-free survival, and distant metastasis-free survival and minimal impact (<2%) on chest wall recurrence over a 10-year period.

We estimated that there would be sufficient chest wall recurrences prevented by adjuvant CWI to lead to a clear gain (7%, HR = 1.225) in overall survival at 10 years, on a baseline survival rate of 56%. However, we observed 10-year survival at approximately 82% and a very low local recurrence rate – the trial results must therefore be interpreted in that context. The 95% CI for the HR for the primary endpoint is compatible with an absolute difference in overall survival (increase or decrease) of up to 3.8%. Although the pre-specified HR of 1.225 is contained within this interval, it represents a far smaller absolute change relative to the observed survival rates than originally estimated. We consider that the original power calculation is no longer informative since breast cancer mortality has fallen considerably^(9)^ since SUPREMO started. Consequently, we consider that the trial provides robust data on the impact of radiotherapy, despite the lower than planned number of events due to the improved survival. Whilst we cannot exclude small positive or negative effects on overall survival, it is biologically implausible that an absolute reduction in chest wall recurrences of less than 2% would translate into a meaningful survival benefit.

Our results contrast with those of the EBCTCG overview^(6)^ (of trials that preceded modern ER/HER2 status testing) showing 10-year reductions in ‘loco-regional recurrence first’ (3.8% vs 20.3%), any first recurrence (34.2% vs 45.7%) and 20-year breast cancer mortality (42.3% vs 50.2%) in 1314 patients with N1 disease, comparable to the 1201 N1 patients in our trial. A likely explanation is higher breast cancer survival,^(9)^ due to advances in multi-disciplinary management, especially diagnostics and systemic therapy.^(23–26)^ A Cochrane review of post-mastectomy radiotherapy indicated that the evidence base from older studies is inapplicable to current practice.^(27)^ Our data challenge the concept that chest wall irradiation should remain a central tenet of locoregional post-mastectomy radiotherapy.

Our results fit with the hypothesis that survival benefit from local therapy increases with more effective systemic therapy but only to a threshold and then declines^(28)^ We suggest that contemporary systemic therapy has breached this threshold and therefore we are observing a decline in benefit from CWI.

Compliance with systemic therapy guidelines was high. The radiotherapy QA program and observed relative reduction in local recurrence – albeit a clinically insignificant absolute reduction of 11 patients in 10 years – provides reassurance that the lack of impact of CWI on overall survival is unlikely to be due inadequate radiotherapy. We believe our pragmatic pathology and treatment guidelines with the broad global spread of trial participants reflect real-world experience and underpin the generalizability of the findings. Since the inclusion period of SUPREMO, advances in systemic therapy have further improved survival, strengthening the rationale for omitting CWI for intermediate-risk patients.

Chest wall recurrence at 10 years was rare in SUPREMO (<3% without CWI), much lower than the 5-15% estimate for intermediate-risk disease at the time of the trial design. Other studies have also reported similarly low incidence of local recurrences with modern multi-modality therapy.^(29, 30)^ The 5-year local recurrence rate in the NSABP-B51/RTOG-1304 trial,^(29)^ which also recruited patients with 1-3 positive nodes, was <1% in unirradiated patients. This is similar to SUPREMO at 5 years. If very few patients experience loco-regional recurrence as a first event without radiotherapy, CWI is unlikely to reduce mortality.^(6)^ The apparent lack of effect of omission of CWI on disease-free and metastatis-free survival is reassuring.

There did not appear to be a differential effect of CWI on overall survival in patients with pN0 or pN1 disease. The 10-year overall survival for patients with pN0 tumors, but with other risk factors, was similar to pN1 patients, justifying inclusion of these intermediate-risk pN0 patients. We confirm grade III, lymphovascular invasion, and larger size confer a similar risk to stage N1 disease.

Subgroup analysis by molecular subtype suggested a worse outcome for TNBC patients following radiotherapy. This finding could be due to low numbers but is consistent with data from NSABP-B51/RTOG-1304, which reported a HR of 2.3 (1.00 – 5.25) for invasive breast cancer recurrence in the irradiated group with TNBC.^(29)^ We speculate that this might be due to a detrimental effect of radiation on immune modulation.

We recognize the recent shift from axillary node clearance to regional nodal irradiation as primary treatment. The EBCTCG overview of trials of regional nodal irradiation shows it achieves a modest reduction in breast cancer mortality in patients with N1 disease,^(31)^ whereas SUPREMO shows no evidence of a survival benefit from the CWI component of post-mastectomy radiotherapy. If regional nodal irradiation is required, modern radiotherapy techniques can deliver homogeneous dose to the lymph node target volumes whilst avoiding the chest wall or reconstructed breast.

Our trial has some limitations. First, SUPREMO was initiated nearly two decades ago. There have been many improvements in practice since then, which have led to lower local recurrence rates. Second, many intermediate risk patients are now treated with neo-adjuvant systemic therapy but this practice was very limited in SUPREMO. The EBCTCG meta-analysis^(32)^ showed no difference in survival between patients treated with adjuvant or neoadjuvant chemotherapy. The NSABP-B51/RTOG-1304 trial^(29)^ included patients with cN1 disease who converted to ypN0 after neo-adjuvant chemotherapy. There was no effect of post-mastectomy radiotherapy in the mastectomy patients on the invasive breast cancer recurrence-free interval. Overall survival at 5 years (approximately 92%) is similar to the survival seen in SUPREMO at the same time point. Third, considerable reductions in axillary surgery have become standard practice. In SUPREMO axillary node clearance was mandatory for N1 disease. Since then, it is apparent that no particular axillary node staging procedure influences overall survival in early breast cancer.^(33)^ Published trials show that axillary node clearance has been replaced by Sentinel Node Biopsy, axillary radiation or no further treatment, including patients treated by neoadjuvant chemotherapy.^(34)^ Finally, the importance of identifying the number of pathologically involved axillary nodes in predicting prognosis is evolving and superseded by a multimodal approach to predict risk of recurrence and death from breast cancer based on a combination of tumor characteristics, axillary imaging and gene expression profiling. The TAILOR RT trial (NCT03488693) for example is utilizing gene expression profiling with Oncotype DX in N1, ER+ and HER2- disease to tailor indications for adjuvant locoregional radiotherapy. The TRANS-SUPREMO tissue archive is available for investigating prognostic and predictive biomarkers.

The low levels of toxicity such as radiation pneumonitis, and paucity of cardiac deaths (≤1%) probably reflects the application of modern radiotherapy techniques. We are aware that radiation-induced cardiac disease and carcinogenesis^(35, 36)^ can present beyond 10 years. If CWI is no longer required in women at intermediate risk, these and other late effects including fibrosis, bone necrosis, muscle and skin atrophy could all be avoided. Our results have implications for breast reconstruction where its use is controversial^(37)^ as some surgeons consider that PMRT is a relative contraindication to reconstruction since radiation increases complication risk and poorer cosmesis. Our findings are likely to reassure surgeons wishing to reconstruct the breast, especially if implants are utilized.

We hope that our results stimulate a re-evaluation of the evidence base for CWI indications. Continuing to recommend CWI, where benefit is marginal and potentially detrimental, may divert limited resources from more effective treatments.^(38)^

In conclusion, SUPREMO provides no evidence to support continued use of adjuvant CWI in most patients with intermediate-risk early breast cancer treated by mastectomy and contemporary adjuvant systemic therapy.

## Supplementary Material

Supplement

## Figures and Tables

**Table 1:** Demographic and clinical characteristics

Characteristic^1^	No Chest Wall Irradiation N = 799	Chest Wall Irradiation N = 808
**Randomizing center**		
UK	586 (73.3%)	582 (72.0%)
EORTC	158 (19.8%)	165 (20.4%)
International	55 (6.9%)	61 (7.5%)
**Age (years)^2^**	55.0 (48.0, 64.0)	54.0 (47.0, 64.0)
**Age group**		
<45 years	121 (15.1%)	130 (16.1%)
45-54 years	267 (33.4%)	285 (35.3%)
55-69 years	309 (38.7%)	283 (35.0%)
70+ years	102 (12.8%)	110 (13.6%)
**Invasive tumor type**		
Ductal, no special type	669 (83.7%)	672 (83.2%)
Lobular carcinoma	78 (9.8%)	75 (9.3%)
Other	51 (6.4%)	57 (7.1%)
Not available	1 (0.1%)	4 (0.5%)
**Histological grade**		
1	42 (5.3%)	57 (7.1%)
2	333 (41.7%)	323 (40.0%)
3	421 (52.7%)	416 (51.5%)
Not available	3 (0.4%)	12 (1.5%)
**Molecular subtypes**		
ER+HER2- or ER+, HER2 unknown	520 (65.1%)	517 (64.0%)
ER+HER2+	95 (11.9%)	110 (13.6%)
ER-HER2+	64 (8.0%)	61 (7.5%)
Triple Negative	84 (10.5%)	91 (11.3%)
Other/ Missing	36 (4.5%)	29 (3.6%)
**TN stage**		
T1N1	226 (28.3%)	246 (30.4%)
T2N0	205 (25.7%)	183 (22.6%)
T2N1	361 (45.2%)	368 (45.5%)
T3N0	3 (0.4%)	4 (0.5%)
T0 or T1, N0 (includes post-NACT)	3 (0.4%)	4 (0.5%)
Other/ missing	1 (0.1%)	3 (0.4%)
**Total number of nodes examined** ^2^	14.0 (9.0, 18.0)	14.0 (10.0, 18.0)
**Total number of nodes involved**		
0	211 (26.4%)	191 (23.6%)
1	312 (39.0%)	330 (40.8%)
2	171 (21.4%)	195 (24.1%)
3	104 (13.0%)	89 (11.0%)
Not available	1 (0.1%)	3 (0.4%)
**Axillary surgery**		
Sentinel node biopsy only	118 (14.8%)	115 (14.2%)
Clearance only	349 (43.7%)	393 (48.6%)
Sentinel or Sample + Clearance	245 (30.7%)	239 (29.6%)
Sample only	40 (5.0%)	31 (3.8%)
Not available	47 (5.9%)	30 (3.7%)
**Immediate breast reconstruction carried out**	80 (10.0%)	95 (11.8%)
**Chemotherapy delivered**		
No	131 (16.4%)	108 (13.4%)
Yes	666 (83.4%)	696 (86.1%)
Not available	2 (0.3%)	4 (0.5%)
**Endocrine Therapy**		
Yes	624 (78.1%)	640 (79.2%)
No	152 (19.0%)	147 (18.2%)
Not available	23 (2.9%)	21 (2.6%)
**Trastuzumab**		
Yes	150 (18.8%)	168 (20.8%)
No	582 (72.8%)	605 (74.9%)
Not available	67 (8.4%)	35 (4.3%)
**Prescribed radiotherapy dose to chest wall**		
40Gy-15F/ 43Gy-16F	1 (0.1%)	444 (55.0%)
50Gy-25F	6 (0.8%)	230 (28.5%)
Other	3 (0.4%)	101 (12.5%)
Not applicable	789 (98.7%)	33 (4.0%)^3^

1 All categorical data shown as Number (%). Percentages are rounded to 1 decimal place (which may not permit exact summation to 100%).

2 Continuous measurements summarized as Median (1^st^ Quartile, 3^rd^ Quartile).

3 This number represents 28 treatment crossovers and 5 patients for whom data were incomplete/ early withdrawals.
