# Supplementary material for: Post-Mastectomy Chest Wall Irradiation Effects on 10-Year Survival in Early Breast Cancer
Source: N Engl J Med. Author manuscript; Available in PMC 2025 Nov 17. (PMC7618363; doi:10.1056/NEJMoa2412225)
Supplement: Supplement [file EMS207966-supplement-Supplement.pdf]

Supplementary Appendix

Table of Contents

1. SUPREMO Trial Collaborators..2
1.1 SUPREMO Trial Investigators..2
United Kingdom..2
EORTC (European Organisation for Research and Treatment of Cancer) 7
International sites 8
1.2 Members of the Trial Management Group 11
1.3 Members of the Trial Steering Committee..13
1.4 Members of Data Monitoring and Ethical Committee 14
1.5 Members of the Writing Committee..15
2. Additional Methodological Information..16
2.1 Inclusion and exclusion criteria of both protocols 16
2.2 Criteria assessed in pathology quality assurance of all trial patients, for eligibility..................19
2.3 Radiotherapy Quality Assurance protocol for all centres participating in the SUPREMO trial...20
3. Supplementary Tables and Graphs..22
3.1 Additional patient and treatment characteristics 22
3.2 Main causes of death 24
3.3 Acute/ late radiation morbidity..25
3.4 Kaplan-Meier plots for primary and selected secondary endpoints in ITT population, stratified by nodal status. 26
3.5 Forest plots for subgroup analyses 27
3.6 Kaplan-Meier plot for overall survival by age subgroups..29
3.7 Kaplan-Meier plot for overall survival by molecular subtypes..30
3.8 Kaplan-Meier plot for chest wall recurrence by molecular subtypes..31
4. Statistical Issues..32
4.1 Software used..32
4.2 Additional statistical methods..32
4.3 Proportional hazards assumptions..33
4.4 Assumption of non-informative censoring..37

1. SUPREMO Trial Collaborators

1.1 SUPREMO Trial Investigators

United Kingdom

(Alphabetical order)

Dr Paul Abram	South Eastern Health and Social Trust
Dr Douglas Adamson	Ninewells Hospital, Dundee
Dr Shahreen Ahmad	Queen Elizabeth Hospital, Woolwich
Dr Hafiz Algurafi<br>Dr Sunil Skaria	Broomfield Hospital, Chelmsford
Dr Abdulla Alhasso	Beatson Oncology Centre
Dr Rozenn Allerton	Russells Hall Hospital, Dudley
Dr Carmel Anandadas	Wigan Royal Albert Edward Infirmary
Dr Charlotte Atkinson<br>Dr Mohini Varughese	Taunton and Somerset NHS Foundation Trust
Dr Amit Bahl	Bristol Haematology & Oncology Centre
Dr Lisa Barraclough	Macclesfield District General Hospital
Professor Peter Barrett-Lee	Velindre Cancer Centre, Cardiff
Dr Urmila Barthakur	Yeovil Hospital, Somerset NHS Foundation Trust
Dr Carolyn Bedi	Western General Hospital, Edinburgh, Borders General Hospital
Dr Mark Beresford	Royal United Hospital Bath
Dr Glen Blackman	University College Hospital, London
Dr David Bloomfield	Royal Sussex County Hospital Brighton, University Hospitals Sussex NHS Foundation Trust
Dr Jo Bowen	Gloucestershire Hospitals NHS Foundation Trust
Dr Lucy Brazil	Guys & St Thomas' Hospital, Queen Mary's Hospital, Sidcup
Dr Jane Brown.<br>Dr Natasha Mithal	Kent and Canterbury Hospital
Alison Brown<br>Dr Jennifer Marshall	St Mary's Hospital, Newport

Dr Andrew Brunt	County Hospital, Stafford
Professor Nigel Bundred	South Manchester University Hospital
Dr Amitabha Chakrabarti<br>Dr Perric Crellin	Poole Hospital (University Hospitals Dorset NHS Foundation Trust)
Dr Amit Chakrabarti	Dorset County Hospital
Dr Hannah Chapman<br>Dr Chin Lim	Tameside General Hospital
Dr Abhro Chaudhuri	Lincoln County Hospital
Dr Abbas Chittalia	Stepping Hill Hospital, Stockport
Dr Mark Churn	Worcestershire Royal Hospital, Kidderminster General Hospital, Alexandra Hospital
Dr Jackie Clarke	Belfast City Hospital
Dr Susan Cleator	Charing Cross Hospital, St Mary's Hospital London
Dr Falalu Danwata	Blackpool Victoria Hospital
Dr Shiroma DeSilva-Minor	Great Western Hospital, Swindon
Dr Amandeep Dhadda	Scarborough General Hospital
Dr Sue Down	James Paget University Hospital
Dr Grainne Dunn<br>Dr Diana Ritchie	Hairmyres Hospital, Victoria ACH, Glasgow
Dr Andrew Eichholz	Wycombe Hospital (BHT), Stoke Mandeville Hospital (BHT),
Dr Rhun Evan<br>Dr Heather McCarty	Belfast City Hospital
Dr Indrajit Fernando	Walsall Hospitals NHS Trust, Birmingham Heartlands Hospital
Dr Jennifer Forrest	North Devon District Hospital, (Royal Devon University Healthcare NHS Foundation Trust (RDUH))
Ghislaine Fraser	Glasgow Royal Infirmary
Dr Judith Fraser	Forth Valley Royal Hospital
Dr Chris Gaffney	Gwent Healthcare NHS Trust
Dr Daljit Gahir<br>Professor Murray Brunt	Royal Stoke Hospital, County Hospital
Dr Konstantinos Geropantas	Norfolk & Norwich University Hospital

Dr Niladri Ghosal<br>Dr Win Soe	Wrexham Maelor Hospital
Dr Andrew Goodman<br>Dr Peter Bliss	Torbay Hospital
Dr Christopher Hamilton<br>Dr Andrew Goodman	Royal Devon and Exeter Foundation Trust, (Royal Devon University Healthcare NHS Foundation Trust), Torbay Hospital
Dr Robert Grieve (deceased)	Walsgrave Hospital, Warwick Hospital
Dr Matthew Griffin	King's Mill Hospital, Sutton in Ashfield
Dr Maher Hadaki	Medway Maritime Hospital
Dr Allison Hall	Countess of Chester Hospital
Dr Monica Haritakis	Scarborough General Hospital
Dr Sarah Harris	King's College Hospital
Olivia Hatcher<br>Dr Conrad Lewanski	Ealing Hospital NHS Trust
Dr Matthew Hatton	Weston Park Hospital, Sheffield
Dr Aisling Hennessy	Dumfries & Galloway
Dr Jonathan Hicks	Wishaw General Hospital
Dr Susan Hignett	MCHT Leighton Hospital
Dr Martin Hogg	Royal Preston Hospital, Chorley District General Hospital, Burnley General Hospital, Royal Blackburn Hospital
Dr Nayyer Iqbal	Nevill Hall Hospital
Dr Kerstie Johnson	Nottingham City Hospital
Dr Julie Jones<br>Dr Jill Bishop	Glan Clwyd Hospital
Dr Rema Jyothirmayi	Kent Oncology Centre, Maidstone Hospital
Dr Claire Kelly<br>Dr Carmel Anandadas	Wigan Royal Albert Edward Infirmary
Dr Anne Kendall	Great Western Hospital, Swindon
Dr Muhammad Khan	New Cross Hospital, Wolverhampton
Dr Sarah Lawless<br>Dr Nicola Storey	James Cook University Hospital
Dr Patricia Lawton	Nottingham City Hospital

Dr Daniela Lee	Newcastle General Hospital, Queen Elizabeth Hospital Gateshead, South Tyneside General Hospital
Dr Conrad Lewanski	Ealing Hospital NHS Trust
Dr Chin Lim	Tameside General Hospital
Dr Imogen Locke	Royal Marsden Hospital, Sutton
Dr Juliette Loncaster	Christie Hospital Manchester, Manchester University NHS Foundation Trust, North Manchester General Hospital
Dr Graeme Lumsden	Crosshouse Hospital
Dr Susan Lupton	George Eliot Hospital
Dr Carol MacGregor	Raigmore Hospital, Inverness
Dr Brian Magee	Salford Royal Hospital
Dr Najibah Mahtab<br>Dr Tony Branson	North Tyneside General Hospital, Wansbeck General Hospital
Dr Zafar Malik	Clatterbridge Centre for Oncology
Dr Sanjana Masinghe	Darlington Memorial Hospital
Dr Gemma McCormick	Medway Maritime Hospital
Dr Marjory McLennan	Dumfries & Galloway, Queen Margaret Hospital, Fife
Dr Ioannis Michalakis<br>Dr Helen Roe	Cumberland Infirmary
Dr Vivek Misra	Royal Oldham Hospital, North Manchester General Hospital, Manchester Foundation Trust
Dr MB Mukesh	Essex County Hospital (Colchester General Hospital)
Dr Shirin Namini	Mid Yorkshire Hospitals NHS Trust
Dr Sarah Needleman	Royal Free Hospital
Dr Lorcan O'Toole	Scunthorpe General Hospital
Dr Mojca Persic	Burton Hospitals NHS Trust, Queens Hospital Burton Derby
Dr Mary Quigley	Barking, Havering & Redbridge Hospitals NHS Trust
Dr Sanjay Raj	Royal Hampshire County Hospital
Dr Pippa Riddle	West Middlesex University Hospital

Dr Diana Ritchie	Victoria ACH, Glasgow
Mr Neil Roberts<br>Dr Sri Kumar	Leeds Teaching Hospitals NHS Trust
Dr Peter Robson	Aintree University Hospital
Dr Helen Roe	Cumberland Infirmary
Dr Martin Rolles	Singleton Hospital, Swansea
Dr Gillian Sadler	Conquest Hospital
Dr Kufre Sampson<br>Dr Dusan Milanovic	Leicester Royal Infirmary
Dr Nihal Shah	Mount Vernon Cancer Centre
Sin Yee (Jackie) Sham	Worthing Hospital, University Hospitals Sussex NHS Foundation Trust
Dr Ravi Sharma	Aberdeen Royal Infirmary
Dr Liz Sherwin	East Suffolk and North Essex NHS Foundation Trust
Dr Peter Simmonds	Southampton University Hospitals NHS Trust
Dr Geraldine Skailes	Furness General Hospital
Dr Win Soe	Wrexham Maelor Hospital
Dr Rajaram Sripadam	Whiston Hospital, St. Helen's
Dr Andrea Stevens	University Hospital Birmingham NHS Foundation Trust
Dr Andrew Stockdale	Solihull Hospital
Dr Isabel Syndikus	Warrington & Halton Hospitals NHS Foundation Trust
Dr Hasina Thandar<br>Dr Anthony Neal	Royal Surrey County Hospital, Ashford & St Peter's Hospital, Chertsey
Dr Emma Thompson	University Hospital of North Tees and Hartlepool
Dr Nicky Thorp	Clatterbridge Centre for Oncology, Royal Liverpool University Hospital
Dr Sunil Upadhyay	Castle Hill Hospital Hull
Professor Jayant Vaidya	The Whittington Hospital, Taunton and Somerset NHS Foundation Trust
Dr Nawaz Walji	Warwick Hospital

Dr Richard Welch	Royal Bolton Hospital
Dr Thomas Wells	Weston General Hospital, Weston-Super-Mare
Dr Sarah Westwell	Eastbourne District General Hospital
Dr Elizabeth Whipp	Frenchay Hospital, North Bristol NHS Trust
Dr Deborah Williamson<br>Dr Chin Lim	Royal Lancaster Infirmary
Dr Virginia Wolstenholme	Barts Health NHS Trust, Whipps Cross
Dr Pamela Woodings	Derbyshire Royal Infirmary
Dr Kathryn Wright	Sunderland Royal Hospital
Dr Frances Yuille	St Johns, Livingston

EORTC (European Organisation for Research and Treatment of Cancer)

(Order of recruitment)

Dr Helen Westenberg (deceased)	Radiotherapie Groep ARTI, Arnhem	Netherlands
Dr Nicola Russell	Antoni Van Leeuwenhoek Hospital - Netherlands Cancer Institute Amsterdam	Netherlands
Dr Izaskun Valduvieco	Hosp. Clinic. Univ., Barcelona	Spain
Dr Marie-Pierre Sunyach<br>Dr Jacques Bonneterre	Centre Léon Bérard, Lyon	France
Dr Elzbieta Senkus-Konefka	Medical University of Gdansk	Poland
Dr John Maduro	University Medical Center Groningen	Netherlands
Dr Stephanie de Boer	Leiden Universitair Medisch Centrum	Netherlands
Dr Victoria Magdalena Reyes Lopez	Hospital General Vall D'Hebron, Barcelona	Spain
Dr Wout Schoevers	Radiotherapie Groep RISO, Deventer	Netherlands
Dr Joeke Tijink-Felderhof<br>Dr Femke Van der Leij	Universitair Medisch Centrum Utrecht	Netherlands
Dr Karolien Verhoeven	Maastro, Maastricht	Netherlands

Dr Karine Peignaux-Casanovas<br>Dr G Truc	Centre Georges-Francois-Leclerc, Dijon	France
Dr Marie-Eve Chand	Centre A. Lacassagne, Nice	France
Dr Wendy Jeanneret	Centre Hospitalier Universitaire Vaudois	Switzerland
Dr Abacioglu Mehmet Ufuk	Marmara University Hospital Istanbul	Turkey
Dr Volker Budach	Charite Universitaetsmedizin Berlin	Germany
Dr. Nina Bijker<br>Dr Geertjan van Tienhoven	Academisch Medisch Centrum, Amsterdam	Netherlands
Dr Raymond Miralbell	Geneva University Hospitals, Cluse Rosarie, Geneva	Switzerland
Dr Roxolyana Abdah-Bortnyak	Rambam Medical Centre, Haifa	Israel
Dr Luc Scheijmans	Dr Bernard Verbeeten Instituut, Tilburg	Netherlands
Dr Wilma Smit	Radiotherapeutisch Instituut Friesland, Leeuwaarden	Netherlands
Dr Anna Brzozowska	Oncology Centre of Lublin	Poland
Dr U. Meier, Dr Daniel Zwahlen	Kantonsspital Winterthur	Switzerland
Dr Gabelle Flandin	Centre hospitalier universitaire de Grenoble	France
Dr Sofia Rivers	Institut Gustave Roussy, Paris	France
Dr Sylvia Giard	Institut Oscar Lambret, Lille	France
Dr Carine Mitine	Hopital de Jolimont	Belgium

International sites

(By country)

Dr Scott Carruthers	Royal Adelaide Hospital	Australia
Dr Anupam Chaudhuri	Riverina Cancer Care Centre	Australia
Dr David Christie	Premion Cancer Care	Australia
Dr Joshua Dass	Sir Charles Gairdner Hospital, Perth, WA	Australia

Dr Steven David	Peter MacCallum Cancer Centre East Melbourne,<br>Peter MacCallum Cancer Centre Moorabbin,<br>Peter MacCallum Cancer Centre Bendigo	Australia
Professor Geoffrey Delaney	Liverpool Hospital Australia	Australia
Dr Brigid Hickey	Radiation Oncology Services Mater Centre (ROMC)	Australia
Dr David Joseph	Sir Charles Gairdner Hospital, Perth, WA	Australia
Dr Sam Leung	ICON Cancer Foundation (Oncology Research Australia, Toowoomba)	Australia
Dr Chen Liu	Peter MacCallum Cancer Centre Box Hill	Australia
Dr Penny Mackenzie	ICON Cancer Foundation	Australia
Dr George Papadatos	Campbelltown Hospital	Australia
Dr Jonathan Ramsay	Radiation Oncology Services, Mater Centre (ROMC), Brisbane	Australia
Dr Vivien Tse	ICON Cancer Foundation	Australia
Dr Xianghui Du	Zhejiang Cancer Hospital	China
Dr Feng-yan Li	Sun Yat-sen University Cancer Center	China
Dr Ping Li	West China Hospital, Sichuan University	China
Professor Yexiong Li	Chinese Academy of Medical Sciences	China
Dr Ping Wang	TianJin Cancer Hospital	China
Dr Dayle Hacking	UPMC Whitfield Cancer Centre	Ireland
Dr Joe Martin	University Hospital Galway	Ireland
Dr Carol McGibney	Cork University Hospital	Ireland
Dr Lorraine Walsh	Mid Western Radiation Oncology Centre, Limerick	Ireland
Dr Hiroji Iwata	Aichi Cancer Center Hospital	Japan
Dr Norikazu Masuda	Osaka National Hospital	Japan
Dr Aruga Tomoyuki	Komagome Hospital	Japan
Dr Hiroyi Yasojima	Osaka National Hospital	Japan

Dr Mathew Seel	Waikato Hospital, Hamilton	New Zealand
Dr Fuh Wong	National Cancer Centre	Singapore

1.2 Members of the Trial Management Group

Professor Ian Kunkler (Chair)	University of Edinburgh	United Kingdom
Dr Nicola Russell (Co-Chief Investigator)	Netherlands Cancer Institute	Netherlands
Dr Marjory MacLennan (Co-Chief Investigator)	Western General Hospital, Edinburgh	United Kingdom
Dr Edwin Aird	Mount Vernon Hospital, Northwood	United Kingdom
Dr Niall Anderson	University of Edinburgh	United Kingdom
Professor John Bartlett	University of Edinburgh	United Kingdom
Jacqueline Burns	SCTRU, PHS, Edinburgh	United Kingdom
Professor John Cairns	London School of Hygiene and Tropical Medicine	United Kingdom
Professor David Cameron	Western General Hospital, Edinburgh	United Kingdom
Dr Peter Canney	Beatson West of Scotland Cancer Centre	United Kingdom
Professor Boon Chua	University of New South Wales, Sydney	Australia
Gaetan de Schaetzen	EORTC	Belgium
Laura Demeulemeester	EORTC	Belgium
Dr Martin Denvir	University of Edinburgh	United Kingdom
Professor Mike Dixon	Western General Hospital, Edinburgh	United Kingdom
Dr Coen Hurkmans	Catharina Ziekenhuis, Eindhoven	Netherlands
Professor Per Karlsson	Göteborg University	Sweden
Marie-Ange Lentz	EORTC	Belgium
Professor Theresa McDonagh	Royal Brompton Hospital, London	United Kingdom
Michael McLaughlin	SCTRU, PHS, Edinburgh	United Kingdom
Fidelis Muturi	SCTRU, PHS, Edinburgh	United Kingdom
Dr David Northridge	Edinburgh Royal Infirmary	United Kingdom
Tammy Piper	University of Edinburgh	United Kingdom

Professor Allan Price	Western General Hospital, Edinburgh	United Kingdom
Kathleen Riddle	SCTRU, PHS, Edinburgh	United Kingdom
Mr Richard Sainsbury	University College, London	United Kingdom
Monika Sobol	Western General Hospital, Edinburgh	United Kingdom
Dr Karen Taylor	University of Edinburgh	United Kingdom
Dr Geertjan van Tienhoven	Academisch Medisch Centrum, Amsterdam	Netherlands
Professor Galina Velikova	St James' University Hospital, Leeds	United Kingdom
Dr Eldo Verghese	Leeds Teaching Hospital	United Kingdom
Alison Walker	Patient representative	United Kingdom
Elizabeth Welch	University of Edinburgh	United Kingdom
Dr Linda Williams	University of Edinburgh	United Kingdom
David Montgomery	University of Edinburgh	United Kingdom
Graeme Patterson	University of Edinburgh	United Kingdom

1.3 Members of the Trial Steering Committee

Professor Barry Hancock (Chair)	University of Sheffield	United Kingdom
Dr Niall Anderson	University of Edinburgh	United Kingdom
Professor John Bartlett	University of Edinburgh	United Kingdom
Carmela Caballero	Breast International Group	Belgium
Dr Peter Canney	Beatson West of Scotland Cancer Centre	United Kingdom
Dr John Graham	Musgrove Park Hospital	United Kingdom
Professor Tim Illidge	University of Manchester	United Kingdom
Dr Richard Jones	Beatson West of Scotland Cancer Centre	United Kingdom
Professor Ian Kunkler	University of Edinburgh	United Kingdom
Fidelis Muturi	SCTRU, PHS, Edinburgh	United Kingdom
Dr Noelle O'Rourke	Beatson West of Scotland Cancer Centre	United Kingdom
Fiach O'Mahony	ACCORD, Edinburgh	United Kingdom
Kathleen Riddle	SCTRU, PHS, Edinburgh	United Kingdom
Dr Morven Roberts	UK Medical Research Council	United Kingdom
Dr Nicola Russell	Netherlands Cancer Institute	Netherlands
Dr Geertjan van Tienhoven	Academisch Medisch Centrum	Netherlands
Professor Galina Velikova	St James' University Hospital	United Kingdom
Alison Walker	Patient representative	United Kingdom

1.4 Members of Data Monitoring and Ethical Committee

Professor Chris Frost (Chair)	London School of Hygiene and Tropical Medicine	United Kingdom
Dr Niall Anderson	University of Edinburgh	United Kingdom
Professor Nicholas James	Institute of Cancer Research, London	United Kingdom
Professor Ian Kunkler	University of Edinburgh	United Kingdom
Fidelis Muturi	SCTRU, PHS, Edinburgh	United Kingdom
Kathleen Riddle	SCTRU, PHS, Edinburgh	United Kingdom
Dr Nicola Russell	Netherlands Cancer Institute, Amsterdam	Netherlands
Professor Paul Symonds	University of Leicester	United Kingdom
Dr Geertjan van Tienhoven	Academisch Medisch Centrum, Amsterdam	Netherlands

1.5 Members of the Writing Committee

Dr Niall Anderson	University of Edinburgh
Professor David Cameron	University of Edinburgh
Professor Michael Dixon	University of Edinburgh
Professor Ian Kunkler	University of Edinburgh
Dr Nicola Russell	Netherlands Cancer Institute
Mr Richard Sainsbury	University College London

2. Additional Methodological Information

2.1 Inclusion and exclusion criteria of both protocols

*indicates criteria broadened or changed in v29 in relation to v27.

Inclusion Criteria protocol version 27

1. pT1 ,N1, M0 unilateral histologically confirmed invasive breast cancer.
2. pT2, N1, M0 unilateral histologically confirmed invasive breast cancer.
3. pT2, N0, M0 unilateral histologically confirmed invasive breast cancer if grade III histology and/ or lymphovascular invasion.
4. Multifocal breast cancer if largest discrete tumor at least 2 cm if N0 and grade III histology and / or lumphovascular invasion [see NB (ii)]
5. If the tumour comprises multiple small adjacent foci of invasive carcinoma then overall maximum dimension taken. This must be greater than 2 cm if N0 (see section 7.2) and grade II histology and/or lymphovascular invasion. [see NB (iii)].
6. Fit for chemotherapy (if indicated), adjuvant endocrine therapy (if indicated) and post-operative radiation.
7. Undergone total mastectomy (with a minimum of 1 mm margin clear of invasive cancer and DCIS) and axillary staging procedure.

7.1 If axillary node positive (1-3 positive nodes) [including micrometastases >0.2 mm - < 2mm]) then an axillary nodal clearance (minimum of 10* nodes removed) should have been performed. Isolated tumour cells do not count as micrometastases.

7.2 Axillary node negative status can be determined on the basis of either axillary clearance or axillary node sampling or sentinel node biopsy.

8. Written, informed consent.

NB (i) patients undergoing immediate breast reconstruction are eligible for inclusion.

NB (ii) Multifocal breast cancer if largest focus conforms to the other eligibility criteria. So if N0 disease the prmary tumour has to have at least one focus size pT2 with grade 3 histology or lymphovascular invasion (criterion 3) or pT1 or pT2 if N1 (criteria 1 and 2).

NB (iii) Criterion 5 is the definition of what is considered pT2 disease for N0 disease for N0 cases (pT1 is allowed if N1). Please also see section 7.2 of the protocol for more detailed explanation.

Exclusion criteria v 27

1. Any pT0, pN0-1, or pT1, N0 or pT3*, pN0-1 or pT4.
2. patients who have 4 or more pathologically involved axillary nodes
3. *Patients who have undergone neo-adjuvant systemic therapy.
4. Previous or concurrent malignancy other than non-melanomatous skin cancer and carcinoma in situ of the cervix.
5. Male
6. *Pregnancy
7. Bilateral breast cancer
8. *Known BRCA1 or BRCA2 carriers
9. Not fit for surgery, radiotherapy or adjuvant systemic therapy
10. Internal mammary nodes visible on sentinel node scintigraphy in the absence of negative histology
11. unable or unwilling to give informed consent

Inclusion criteria protocol version 29 August 2010
1.1 Stage II histologically confirmed unilateral breast cancer following mastectomy including the following pTNM stages:<br>• pT1N1M0<br>• pT2N1M0<br>• pT2N0M0 if grade III histology and/or lymphovascular invasion<br>• *pT3N0M0. If the tumour area comprises multiple small adjacent foci of invasive carcinoma then overall maximum dimension is taken to determine the size for T staging (see section 7.2.2 for a more detailed explanation). Multifocal or multicentric tumours can be included (pT1m; pT2m; pT3m). The size of the largest tumour focus determines the T stage classification. See section 7.2.1).<br><br>1.2 Stage II histologically confirmed unilateral breast cancer following neoadjuvant systemic therapy and mastectomy, if the original clinical stage was cT1-2cN0-1M0 or cT1-2pN1(sn)M0 and with the following (ypTNM) stages after neoadjuvant systemic therapy:<br>• *ypT1pN1M0<br>• *ypT2pN1M0<br>• *ypT2pN0M0 if grade III histology and/or lymphovascular invasion.<br>• *ypT0pN0 or ypT1pN0 or ypT0pN1 (pathological complete remission, or near complete remission).<br>• * ypT3N0M0, if original clinical staging was cT1-3cN0 M0 or cT1-3pN0 (sn) M0.<br><br>1.3 *Unilateral invasive breast cancer that conforms to the initial clinical staging of criterion 1, but has been down-staged by neoadjuvant systemic therapy to ypT0 pN0 or ypT1pN0 or ypT0pN1 (pathological complete remission, or near complete remission). If tumour stage cT3 or ypT3, then nodal status must be N0 both before and after neoadjuvant systemic therapy.<br><br>2. Undergone total mastectomy (with minimum of 1 mm clear margin of invasive cancer and DCIS) and axillary staging procedure.<br><br>3.1 *If axillary node positive (1-3 positive nodes [including micrometastases >0.2mm-≤2mm]) then an axillary node clearance (minimum of 8* nodes removed) should have been performed. Isolated tumour cells do not count as micrometastases.<br><br>3.2 Axillary node negative status can be determined on the basis of either axillary clearance or axillary node sampling or sentinel node biopsy.<br><br>3.3 *Sentinel nodes identified in the internal mammary chain are considered pN1b or pN1c if histologically proven. Patients can be included in the trial with microscopic metastasis in the internal mammary chain detected by sentinel node biopsy, if not more than 3 tumour positive nodes in axillary lymph nodes. If not biopsied, internal mammary chain sentinel nodes are considered tumour negative for staging.<br><br>3.4 *Before neoadjuvant systemic therapy, axillary ultrasound is advised. Abnormal axillary nodes based on imaging (mammogram or ultrasound) should be sampled by guided needle sampling or core biopsy. Where axillary ultrasound is normal, negative axillary node status does not require histological confirmation before starting neoadjuvant systemic therapy. Positive, or negative, nodal status may also be determined by sentinel node biopsy before start of neoadjuvant therapy.<br><br>4. Fit for adjuvant or neoadjuvant chemotherapy (if indicated), adjuvant or neoadjuvant endocrine therapy (if indicated) and postoperative irradiation.<br><br>5. Written, informed consent.

Additional explanation for the inclusion criteria:

1. Patients undergoing immediate breast reconstruction are eligible for inclusion.
2. *Patients who are carriers of known pathological mutations in BRCA1 or BRCA2 genes are eligible for inclusion.
3. *Neoadjuvant systemic therapy: 3.1 Patients who have undergone mastectomy after neoadjuvant systemic therapy are eligible for inclusion. For determination of tumour stage and nodal involvement, please see Section 7.3. 3.2 Tumour grade, hormone receptor status and Her-2 receptor status (or HER gene amplification) should be determined on a core biopsy taken before the start of neoadjuvant systemic therapy. Lymphovascular invasion may be assessed on both the core biopsy and post treatment excision. 3.3. T2 tumours that are cN0 and remain ypN0 after neoadjuvant systemic therapy can only be included if grade III histology and / or lymphovascular invasion.
3.4 T3 tumours can only be included if N0 both before and after neoadjuvant systemic therapy (cN0, pN0(sn), ypN0).

Exclusion criteria version 29

1. Any pT0pN0-1 or pT1pN0 tumours after primary surgery.
2. Any pT3pN1 or pT4 tumours. Initial stage cT3cN1 or pN1(sn) or cT4 in patients receiving neoadjuvant systemic therapy cannot be included, even if downstaging has occurred and the pathological ypT and N stage is lower.
3. Patients who have 4 or more pathologically involved axillary nodes. For the purpose of this study protocol, nodal scarring after neoadjuvant systemic therapy will be considered as evidence of previous pathological nodal involvement and count towards the total number of involved axillary nodes.
4. Past history or concurrent diagnosis of ductal carcinoma in situ (DCIS) of the contralateral breast, unless treated by mastectomy. Previous DCIS of the ipsilateral breast if treated with radiotherapy (i.e. previous DCIS treated by conservation surgery not followed by radiotherapy would be considered eligible).
5. Bilateral breast cancer. However, patients who have undergone a prophylactic contralateral mastectomy can be included, if the breast was pathologically free of invasive tumour.
6. Previous or concurrent malignancy other than non-melanomatous skin cancer and carcinoma in situ of the cervix. For previous DCIS see criterion 4.
7. Male.
8. *Pregnancy, at the time of radiotherapy treatment.
9. Not fit for surgery, radiotherapy or adjuvant systemic therapy.

2.2 Criteria assessed in pathology quality assurance of all trial patients, for eligibility

Based on the pathology reports for each patient.

Conducted by IHK, NSR GvT

Administrative	Primary tumor characteristics	Nodal characteristics	Surgical	Eligibility
Patient trial number	Multifocal?	Extent of axillary surgery<br>SNP/ Sample/ ANC or combination	laterality	Reviewer comments
Site / centre	Size of invasive tumor	# nodes examined	Margin > 1 mm	Eligible
1st Path report number	Histological type	# nodes involved		Non eligible
2nd Path report number	B-R Scores for: mitoses, nuclei and tubules			Ineligible major violation
3rd Path report number	Grade			Ineligible minor violation
Date checked	LVI			
Reviewer	Oestrogen status			
	Progesterone status			
	HER2 –IHC test			
	Her2-amplification test			

2.3 Radiotherapy Quality Assurance protocol for all centres participating in the SUPREMO trial

Prior to centers entering any patients into the trial

i. All centres will be required to complete a QA questionnaire detailing:- treatment techniques, availability of equipment, simulation procedures, immobilization, protocol for gaps and breaks in treatment, center policy on use of bolus etc. This can be downloaded from the web site www.rttrialsqa.org.uk . A staff questionnaire giving contact details who should be contacted with queries by the QA team should also be completed. For centres in the UK who have not previously completed the baseline questionnaire, this should also be completed. For centres in the EORTC the facilities questionnaire should be completed www.italianhosp-haifa.org/protected_Facility%20Questionnaie.htm

ii. All centres will be required to produce treatment plans (dummy runs) for 2 sample patient outlines. DICOM files of the patients CT scans are available for centres who would normally plan these patients using CT. Alternatively hard copy outlines of 3 selected patients are available from the QA team. The patients should be planned as closely as possible to the method that would be used for patients in the trial and the plans submitted either electronically or hard copy for central review. Centres using electronic submission should send the data on CD and provide either

   a. DICOM-RT export to include dose, data image, structures outlined and rtplan. Please include image data as this is sometimes re-interpolated by the planning system.
   b. RTOG export including at least the images and the dose.
   c. Plato planning system may also export data electronically please contact us for details of files to send.

iii. Centres should provide evidence of an external audit of treatment machine output for at least 1 photon energy (if centers is planning to use electrons then an electron audit should also have been performed) within the last 3 years e.g. ESTRO equal program or IAEA. In the UK interdepartmental audit group visits are also acceptable. A photocopy of the results or summary letter should be attached.

Preferably before entering any patients into the trial but within three months of a center entering patients into the trial

i. Centres will be required to input a series of geometrical blocks into the planning system to assess the algorithm for scatter compensation, unless an audit involving a chest all shaped phantom has been performed on their planning system (e.g. START).

ii. For centres using electrons comparison of measurements vs calculated (either by hand or planning system) for normal and oblique incidence will be required (protocol not yet written but to include e.g. depth of 90% isidose and an angle of incidence of 45°, check for output factors for large applicators at energies used to treat chest wall patients).

During the trial

i. A subset of patients (approximately 1 in 10) will be required to undergo TLD measurements, probably using TLD supplied by the UK trials centre. This is currently untested outside the UK and centres may have to ask to supply information abut their own in vivo measurements.

ii. The QA team will collect the plans, together with verification images, for the first 5 patients in the treatment arm from each centre. Before any copies of treatment records are sent fo central review, any personal identifiers patient name, medical record/ hospital ID) must have been removed and replaced with the patient's trial registration number and initials. If you do not curenty have software for this please contact us before sending patients' data. For centres only able to submit paper printout 3 slices should be sent if possible, central axis plus a slice 3 cm inside the superior and inferior border (or your current clinical practice, but preferably close to this). Please ensure that at least the following isodoses, 50%, 80%, 90%, 95%, 100%, 105%, 107%, 110%, 115% are included.

Cardic study patients

i. For centres participating in the cardiac study, all treatment plans, simulator images and verification images should be sent to the QA team, preferably electronically. If hard copy submission is necessary please contact us so we can check the most appropriate data is submitted.

EORTC all dummy run data to be sent to Coen Hurkmans initially. Patient data to UK trials office (see below)

Coen.hurkmans@cathaina-ziekenhuis.nl

UK/ANZ all data to be sent to Karen Venables/ Fiona Butler initially. Also EORTC clinical patient data.

trials@rttrialsqa.org.uk

3. Supplementary Tables and Graphs

3.1 Additional patient and treatment characteristics

Characteristic[1]	No Chest Wall Irradiation N = 799	Chest Wall Irradiation N = 808
**Estrogen receptor status**		
Positive	615 (77.0%)	627 (77.6%)
Negative	177 (22.2%)	172 (21.3%)
Not available	7 (0.9%)	9 (1.1%)
**Progesterone receptor status**		
Positive	413 (51.7%)	406 (50.2%)
Negative	240 (30.0%)	257 (31.8%)
Not available	146 (18.3%)	145 (17.9%)
**HER-2 positive**		
Yes	160 (20.0%)	172 (21.3%)
No	557 (69.7%)	556 (68.8%)
Not available	82 (10.3%)	80 (9.9%)
**Triple negative**		
Yes	84 (10.5%)	91 (11.3%)
No	684 (85.6%)	692 (85.6%)
Not available	31 (3.9%)	25 (3.1%)
**Anthracycline**		
Yes	620 (77.6%)	642 (79.5%)
No	45 (5.6%)	52 (6.4%)
Not available	134 (16.8%)	114 (14.1%)
**Taxane**		
Yes	384 (48.1%)	421 (52.1%)
No	281 (35.2%)	273 (33.8%)
Not available	134 (16.8%)	114 (14.1%)
**Duration of chemotherapy (weeks)[2]**	15.3 (15.0, 17.9)	15.3 (15.0, 17.9)
Not available	134 (17%)	115 (14%)
**Timing of chemotherapy relative to mastectomy**		
Before	6 (0.8%)	11 (1.4%)
After	659 (82.5%)	681 (84.3%)
Both	1 (0.1%)	4 (0.5%)
None	131 (16.4%)	108 (13.4%)
Not available	2 (0.3%)	4 (0.5%)
**Treatment given to Medial SCF**	12 (1.5%)	97 (12.0%)
**Treatment given to IMC**	7 (0.9%)	12 (1.5%)
**Radiotherapy technique**		

Characteristic[1]	No Chest Wall Irradiation<br>N = 799	Chest Wall Irradiation<br>N = 808
Photon therapy	4 (0.5%)	606 (75.0%)
Electron therapy	1 (0.1%)	120 (14.9%)
Combination	9 (1.1%)	40 (5.0%)
Not irradiated or missing	785 (98.2%)	42 (5.2%)
**Bolus**		
Bolus - Scar only	0 (0.0%)	61 (7.5%)
Bolus - Whole chest wall	6 (0.8%)	294 (36.4%)
No Bolus	9 (1.1%)	420 (52.0%)
Not irradiated or missing	784 (98.1%)	33 (4.1%)
**Radiotherapy completed according to Protocol**		
Yes	11 (1.4%)	751 (92.9%)
No	5 (0.6%)	29 (3.6%)
Not irradiated or missing	783 (98.0%)	28 (3.5%)
**Death from any cause**	145 (18.1%)	150 (18.6%)

[1] All categorical data shown as Number (%). Percentages are rounded to 1 decimal place (which may not permit exact summation to 100%).

[2] Summarized as Median (1st Quartile, 3rd Quartile).

[3] This number represents 28 treatment crossovers and 5 patients for whom data were incomplete/ early withdrawals.

3.2 Main causes of death

Characteristic	No Chest Wall Irradiation<br>N = 145	Chest Wall Irradiation<br>N = 150
**Main cause of death[1]**		
Breast Cancer	95 (65.5%)	99 (66.0%)
Lung Cancer	7 (4.8%)	7 (4.7%)
Other Cancer	11 (7.6%)	12 (8.0%)
Cardiac	8 (5.5%)	6 (4.0%)
Cerebrovascular	2 (1.4%)	3 (2.0%)
Respiratory Disease	9 (6.2%)	5 (3.3%)
Sepsis	2 (1.4%)	3 (2.0%)
Other	11 (7.6%)	15 (10.0%)

[1] Reported as Number (%). Percentages are rounded to 1 decimal place (which may not permit exact summation to 100%).

3.3 Acute/ late radiation morbidity

**Characteristic**[1]	**No Chest Wall Irradiation** N = 799	**Chest Wall Irradiation** N = 808
**Maximum lung toxicity recorded (up to 2 years)**		
0	771 (97.3%)	756 (94.1%)
1	16 (2.0%)	34 (4.2%)
2	5 (0.6%)	9 (1.1%)
3	0 (0.0%)	3 (0.4%)
4	0 (0.0%)	1 (0.1%)
Missing	7 (0.9%)	5 (0.6%)
**Lung toxicity of 2 or more**		
Absent	787 (99.4%)	790 (98.4%)
Present	5 (0.6%)	13 (1.6%)
Missing	7 (0.9%)	5 (0.6%)
**Maximum heart toxicity (all values to 10 years)**		
0	751 (94.2%)	753 (93.4%)
1	26 (3.3%)	26 (3.2%)
2	10 (1.3%)	12 (1.5%)
3	8 (1.0%)	14 (1.7%)
4	2 (0.3%)	1 (0.1%)
Missing	2 (0.3%)	2 (0.2%)
**Heart toxicity of 3 or more**		
Absent	787 (98.7%)	791 (98.1%)
Present	10 (1.3%)	15 (1.9%)
Missing	2 (0.3%)	2 (0.2%)
**Maximum bone toxicity (all values to 10 years)**		
0	693 (88.2%)	679 (85.3%)
1	55 (7.0%)	65 (8.2%)
2	34 (4.3%)	45 (5.7%)
3	4 (0.5%)	3 (0.4%)
4	0 (0.0%)	4 (0.5%)
Missing	13 (1.6%)	12 (1.5%)
**Bone toxicity of 3 or more**		
Absent	782 (99.5%)	789 (99.1%)
Present	4 (0.5%)	7 (0.9%)
Missing	13 (1.6%)	12 (1.5%)

[1] Reported as Number (%). Percentages are rounded to 1 decimal place (which may not permit exact summation to 100%).

3.4 Kaplan-Meier plots for primary and selected secondary endpoints in ITT population, stratified by nodal status.

Note that for clarity, the vertical scale is truncated to survival probabilities between 0.7 and 1.0 and the horizontal scale excludes follow-up longer than 10 years. A: Overall survival. B: Chest wall recurrence. C: Distant metastasis-free survival. D: Disease-free survival. HR is for the main effect of nodal status (N1 relative to N0), adjusted for treatment and center.

A

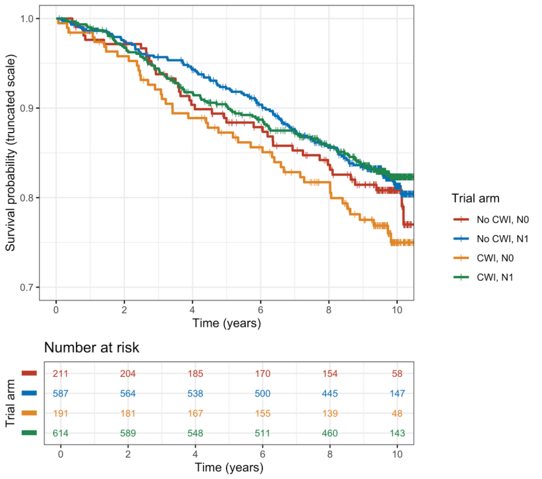

HR 0.82, 95% CI (0.63 to 1.05)

B

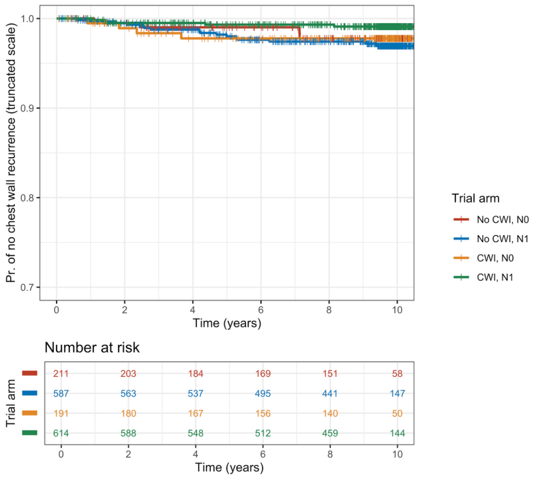

HR 0.91, 95% CI (0.40 to 2.05)

C

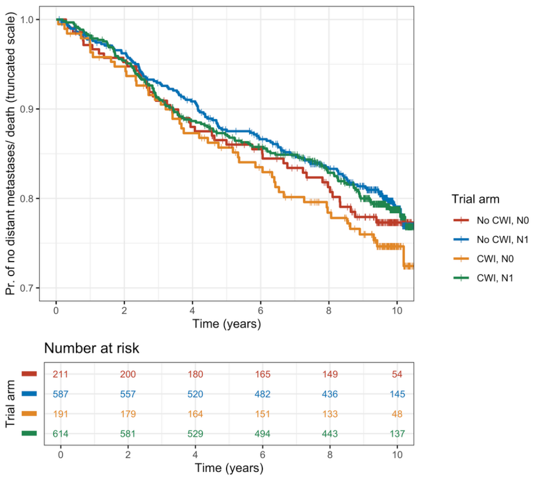

HR 0.87, 95% CI (0.69 to 1.10)

D

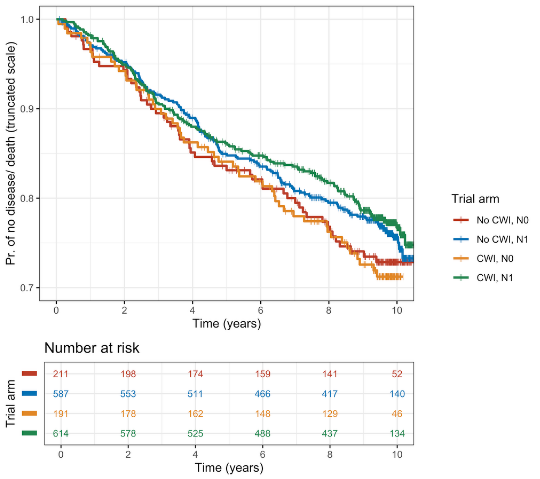

HR 0.84, 95% CI (0.67 to 1.05)

3.5 Forest plots for subgroup analyses

Figure 3.5A: Forest plot of HR for randomized treatment (CWI versus no CWI), adjusted for center, for the endpoint of chest wall recurrence-free survival within age, nodal status and molecular subtype[1] subgroups. For comparison, the original HR for randomised treatment (stratified only by center) is provided at the bottom of the plot.

Subgroups	N (no CWI)	Events (no CWI)	N (CWI)	Events (CWI)	HR (95% CI)
Age group (years)					
<45 years	121	7	130	4	0.57 (0.17 to 1.97)
45-54 years	267	4	285	2	0.46 (0.09 to 2.53)
55-69 years	309	6	283	3	0.56 (0.14 to 2.23)
70+ years	102	3	110		0.00 (0.00 to Inf)
Nodal Status					
N0	211	4	191	4	1.13 (0.28 to 4.54)
N1-3	587	16	614	5	0.30 (0.11 to 0.82)
Molecular subtypes					
ER+HER2-	460	10	455	4	0.40 (0.13 to 1.28)
ER+HER2+	95	3	110		0.00 (0.00 to Inf)
ER-HER2+	64	1	61		0.00 (0.00 to Inf)
Triple Negative	84	4	91	4	1.03 (0.26 to 4.12)
Overall model					
HR for CWI vs. No CWI	799	20	808	9	0.45 (0.20 to 0.99)

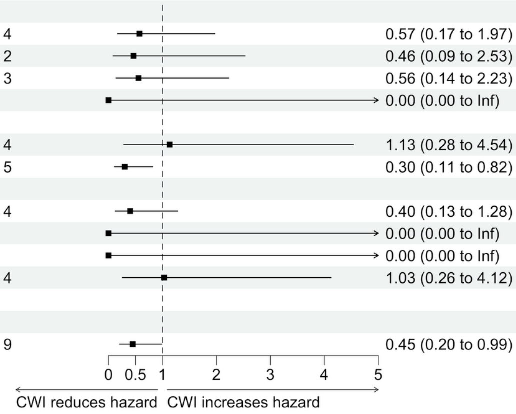

Figure 3.5B: Forest plot of HR for randomized treatment (CWI versus no CWI), adjusted for center, for the endpoint of locoregional recurrence within age, nodal status and molecular subtype[2] subgroups. For comparison, the original HR for randomised treatment (stratified only by center) is provided at the bottom of the plot.

Subgroups	N (no CWI)	Events (no CWI)	N (CWI)	Events (CWI)	HR (95% CI)
Age group (years)					
<45 years	121	13	130	7	0.52 (0.21 to 1.31)
45-54 years	267	12	285	5	0.39 (0.14 to 1.10)
55-69 years	309	8	283	9	1.25 (0.48 to 3.24)
70+ years	102	3	110	1	0.29 (0.03 to 2.79)
Nodal Status					
N0	211	8	191	7	1.00 (0.36 to 2.77)
N1-3	587	28	614	15	0.51 (0.27 to 0.96)
Molecular subtypes					
ER+HER2-	460	18	455	11	0.61 (0.29 to 1.29)
ER+HER2+	95	4	110	1	0.19 (0.02 to 1.70)
ER-HER2+	64	2	61		0.00 (0.00 to Inf)
Triple Negative	84	6	91	8	1.35 (0.47 to 3.91)
Overall model					
HR for CWI vs. No CWI	799	36	808	22	0.61 (0.36 to 1.03)

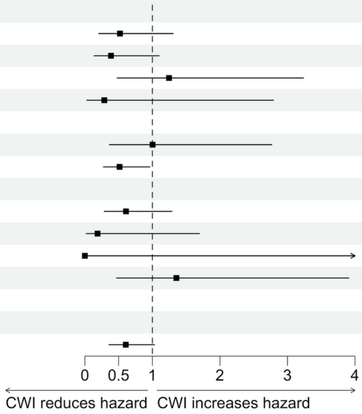

[1] The treatment parameters for the oldest age group and the ER+HER2+ and ER-HER2+ subtypes were not estimable, and the HR shown here are not reliable.
[2] The treatment parameter for the ER-HR2+ subgroup was not estimable, and the HR shown here is not reliable.

Figure 3.5C: Forest plot of HR for randomized treatment (CWI versus no CWI), adjusted for center, for distant metastasis-free survival within age, nodal status and molecular subtype subgroups. For comparison, the original HR for randomised treatment (stratified only by center) is provided at the bottom of the plot.

Subgroups	N (no CWI)	Events (no CWI)	N (CWI)	Events (CWI)	HR (95% CI)
Age group (years)					
<45 years	121	23	130	23	0.98 (0.55 to 1.75)
45-54 years	267	39	285	53	1.35 (0.89 to 2.05)
55-69 years	309	62	283	61	1.06 (0.75 to 1.52)
70+ years	102	42	110	39	0.77 (0.50 to 1.21)
Nodal Status					
N0	211	47	191	49	1.16 (0.78 to 1.74)
N1-3	587	119	614	126	1.02 (0.79 to 1.31)
Molecular subtypes					
ER+HER2-	460	95	455	89	0.95 (0.71 to 1.27)
ER+HER2+	95	15	110	21	1.22 (0.63 to 2.37)
ER-HER2+	64	14	61	13	0.98 (0.45 to 2.17)
Triple Negative	84	18	91	33	1.93 (1.09 to 3.44)
Overall model					
HR for CWI vs. No CWI	799	166	808	176	1.06 (0.86 to 1.31)

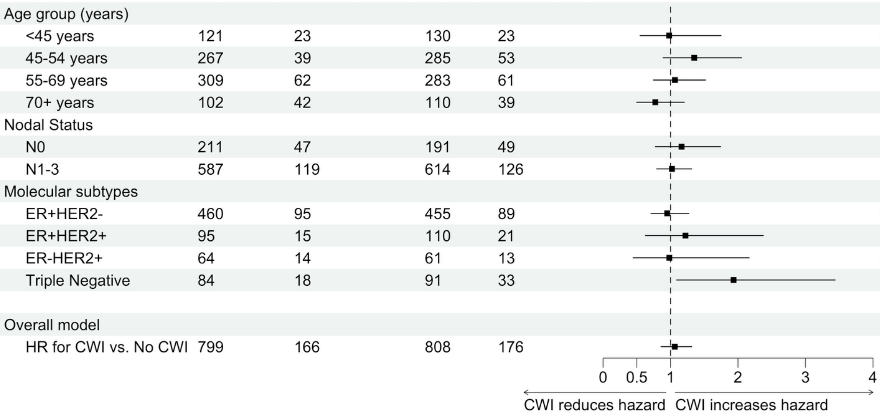

Figure 3.5D: Forest plot of HR for randomized treatment (CWI versus no CWI), adjusted for center, for disease-free survival within age, nodal status and molecular subtype subgroups. For comparison, the original HR for randomised treatment (stratified only by center) is provided at the bottom of the plot.

Subgroups	N (no CWI)	Events (no CWI)	N (CWI)	Events (CWI)	HR (95% CI)
Age group (years)					
<45 years	121	31	130	26	0.80 (0.47 to 1.35)
45-54 years	267	47	285	59	1.22 (0.83 to 1.80)
55-69 years	309	73	283	66	0.97 (0.70 to 1.36)
70+ years	102	45	110	41	0.76 (0.50 to 1.17)
Nodal Status					
N0	211	56	191	55	1.07 (0.74 to 1.56)
N1-3	587	140	614	136	0.93 (0.73 to 1.17)
Molecular subtypes					
ER+HER2-	460	111	455	101	0.92 (0.70 to 1.20)
ER+HER2+	95	17	110	21	1.04 (0.55 to 1.97)
ER-HER2+	64	18	61	15	0.82 (0.40 to 1.67)
Triple Negative	84	22	91	33	1.53 (0.89 to 2.63)
Overall model					
HR for CWI vs. No CWI	799	196	808	192	0.97 (0.79 to 1.18)

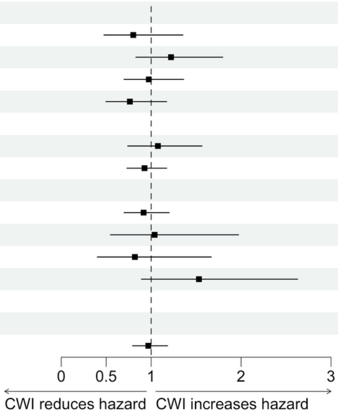

3.6 Kaplan-Meier plot for overall survival by age subgroups

Note that the horizontal axis is truncated at 10 years for clarity.

Patients in the age group >70 years had poorer OS compared to the other age groups (<45, 45-54, 55-69), with an increased risk of death in the oldest age group relative to the youngest (HR 2.74; 95% CI, 1.85 to 4.04), but this was not affected by CWI allocation.

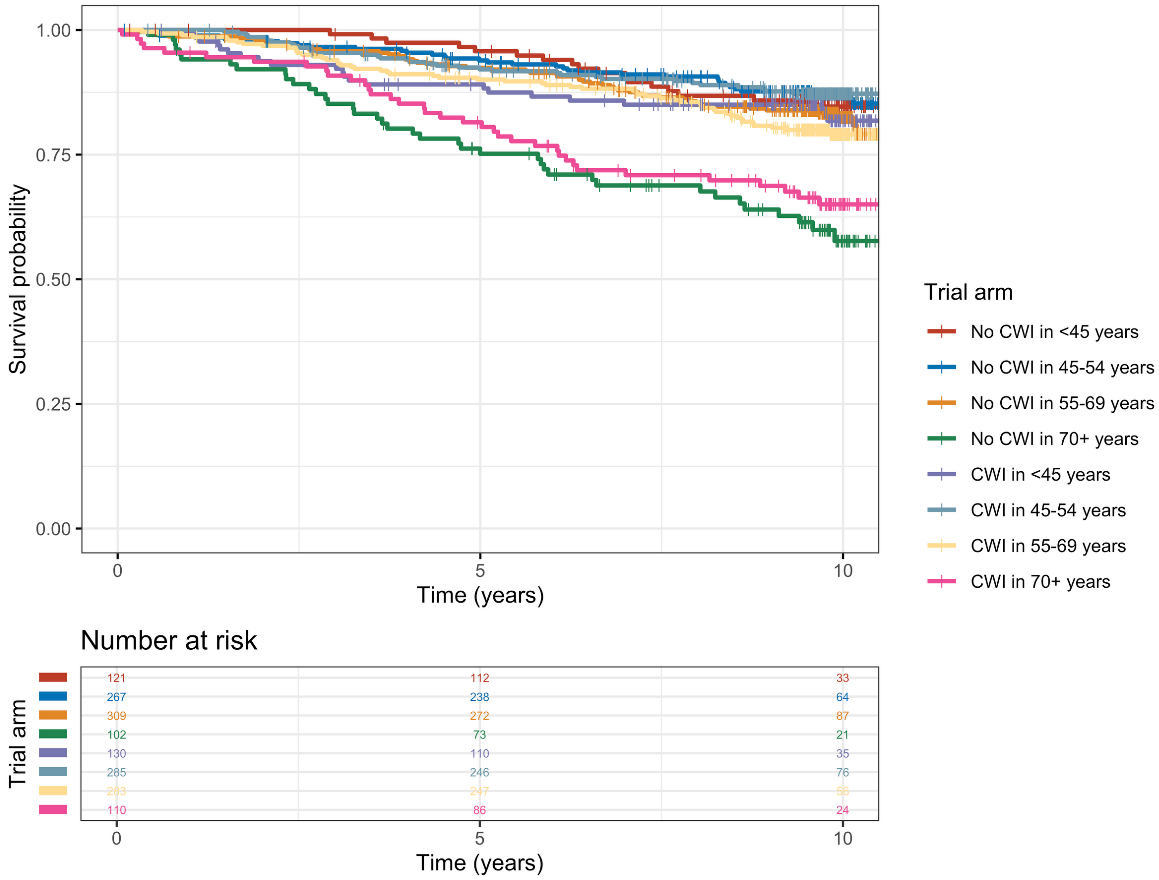

3.7 Kaplan-Meier plot for overall survival by molecular subtypes

Note that the vertical axis is truncated to lie between 0.5 and 1.0 and the horizontal axis is truncated at 10 years for clarity.
A: Complete Kaplan-Meier plot for all 4 subtypes.
B: Sub-plot for triple negative subtype only.

Triple-negative breast cancer (TNBC) had an increased hazard of mortality relative to the ER+HER2 negative reference group (HR 1.895; 95% CI, 1.365 to 2.531).

A

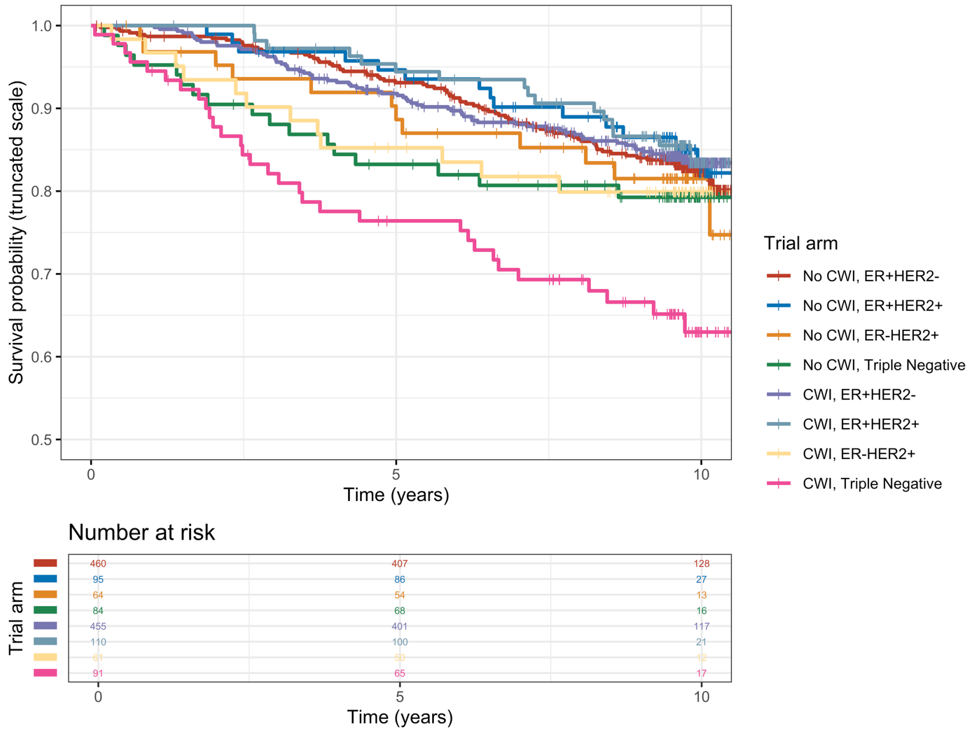

B

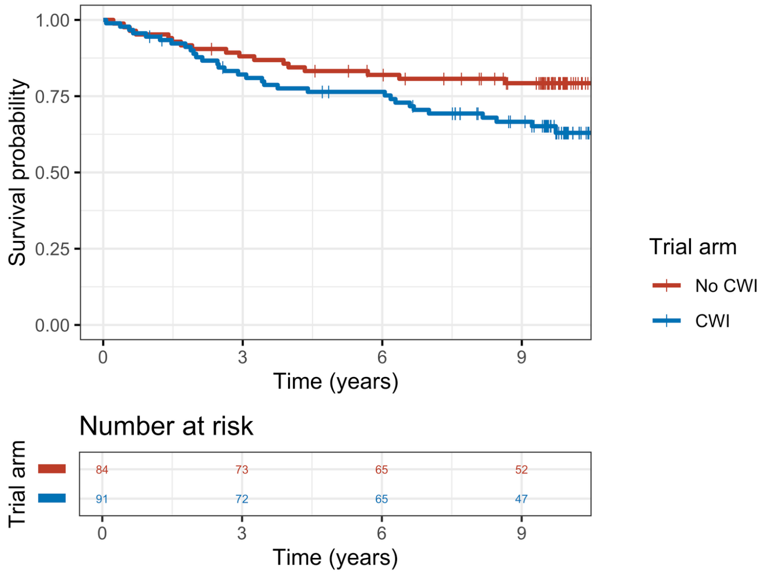

3.8 Kaplan-Meier plot for chest wall recurrence by molecular subtypes

Note that the vertical axis is truncated to lie between 0.9 and 1.0 and the horizontal axis is truncated at 10 years for clarity.
A: Complete Kaplan-Meier plot for all 4 subtypes.
B: Sub-plot for triple negative subtype only.

Patients with TNBC had an increased hazard of chest wall recurrence relative to ER+ HER2 negative cancers (HR 3.45; 95% CI, 1.45 to 8.24), but this was not reduced by CWI.

A

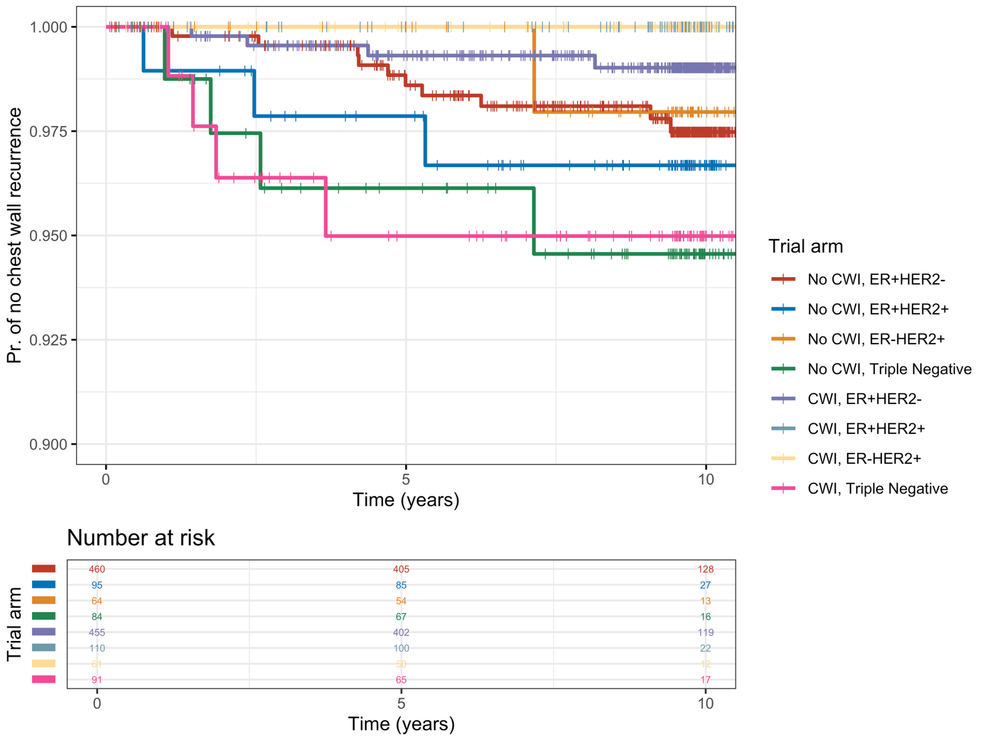

B

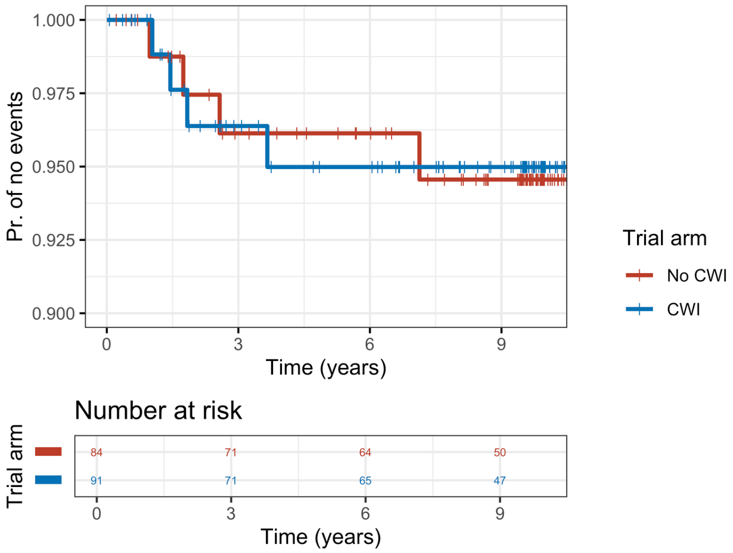

4. Statistical Issues

4.1 Software used

Analyses were conducted with R version 4.4.2[3], using RStudio 2024.09.0-375. Libraries used included

- tidyverse[4]
- survival[5]
- survminer[6]
- consort[7]
- ggsci[8]
- gtsummary[9]
- forestploter[10]

4.2 Additional statistical methods

The following definitions of events for analyses of distant metastasis-free survival (DMFS) and disease-free survival (DFS) were used. An event was recorded if one or more of the categories below were identified for the patient:

DMFS - first occurrence of a distant metastasis

- death

DFS - first locoregional recurrence

- first distant metastasis

- first contralateral breast cancer (new breast primary)

- death

---

[3] R Core Team (2024). R: A Language and Environment for Statistical Computing. R Foundation for Statistical Computing, Vienna, Austria. <https://www.R-project.org/>
[4] Wickham H, Averick M, et al (2019). "Welcome to the tidyverse." _Journal of Open Source Software, 4 (43), 1686. doi:10.21105/joss.01686 <https://doi.org/10.21105/joss.01686>.
[5] Therneau T (2024). A Package for Survival Analysis in R. R package version 3.7-0, https://CRAN.R-project.org/package=survival;
Therneau, TM & Grambsch, PM (2000). Modeling Survival Data: Extending the Cox Model. Springer, New York. ISBN 0-387-98784-3.
[6] Kassambara A, Kosinski M, Biecek P (2024). survminer: Drawing Survival Curves using 'ggplot2'. R package version 0.5.0, <https://CRAN.R-project.org/package=survminer>.
[7] Dayim A (2024). consort: Create Consort Diagram. R package version 1.2.2, <https://CRAN.R-project.org/package=consort>.
[8] Xiao N (2024). ggsci: Scientific Journal and Sci-Fi Themed Color Palettes for 'ggplot2'. R package version 3.2.0, <https://CRAN.R-project.org/package=ggsci>.
[9] Sjoberg DD, Whiting K, Curry M, Lavery JA, Larmarange J. Reproducible summary tables with the gtsummary package. The R Journal 2021;13:570–80. https://doi.org/10.32614/RJ-2021-053.
[10] Dayimu A (2025). forestploter: Create a Flexible Forest Plot. R package version 1.1.3, <https://CRAN.R-project.org/package=forestploter>.

Kaplan-Meier plots for the main endpoints reported in the paper are drawn unadjusted for the center/ geographical effects, thus represent only unadjusted treatment differences. On advice from the Data Monitoring and Ethical Committee, individual centers were grouped into 3 geographical regions (UK, continental Europe and rest of the world) to reduce the possibility of overparameterization of the final models.

Primary and secondary analyses using the Cox proportional hazards model proceeded by fitting a full model containing treatment arm and center main effects plus the interaction between the two. None of those interactions were found to be statistically significant (defined as $p<0.05$), and so each model presented is the main effects-only model for the relevant endpoint.

For the analyses of treatment effect within age, nodal status and molecular subtype subgroups, models containing treatment arm, center and subgroup main effects plus treatment by subgroup and center by subgroup pairwise interactions were taken as the initial model fit. No center by subgroup terms were statistically significant ($p<0.05$), and were therefore removed in each case. Treatment by subgroup interaction terms were of interest in these analyses, and therefore recorded for reporting.

For the analyses of acute/ late morbidity, a simplified analysis was undertaken (as suggested in the Statistical Analysis Plan, SAP), since higher values of lung, heart and bone toxicity seemed relatively rare in the trial population. For lung toxicity, we considered an occurrence of grade 2 toxicity or more within 2 years of radiotherapy treatment to represent an endpoint of “toxicity present”. For heart and bone toxicity, we considered occurrence of grade 3 or more at any point within the 10 years’ follow-up to represent a “toxicity present” endpoint. Analysis proceeded by fitting a logistic regression model for each endpoint, in terms of treatment and centre. Given the low numbers of endpoints, a treatment by center interaction term was omitted.

No multiplicity adjustment was specified in the SAP, and thus all confidence interval widths are reported as originally calculated (interval widths/ confidence levels are not adjusted for multiplicity).

An early interim analysis (originally scheduled for the observation of 300 events) was considered by the IDMC in 2019-20 but the impact of the COVID-19 pandemic in reducing staff access to the trial administrative centre, the lack of funding to conduct such an analysis and the proximity of the completion of the recruitment period precluded it. The IDMC documented their decision no longer to recommend an interim analysis.

4.3 Proportional hazards assumptions

The proportional hazards assumption for each variable in each Cox model was checked by a score test for zero slope in plots of Schoenfeld residuals against study time (via the *cox.zph()* function from the R *survival* package). Table S4 below reports the p-values obtained for the primary and secondary endpoint analyses, while Table S5 reports equivalently for the subgroup analyses for age group, nodal status and molecular subtype.

Table S4: P-values for score tests of the proportional hazards assumptions for the primary and secondary analysis models

Analysis endpoint	Score test p-value	
	**Randomised Treatment**	**Centre**
Overall survival	0.057	0.075
Chest wall recurrence	0.109	0.250
Regional recurrence	***0.011***	0.734
Metastasis-free survival	0.421	0.265
Disease-free survival	0.856	0.573

For the randomised treatment effect in the analysis of regional recurrence, the score test p-value showed moderate evidence of a loss of proportionality. The Schoenfeld residual plot for this parameter is shown below in Figure S9, which is suggestive that the reduction in hazard associated with the calculated HR may mainly derive from later time points only. For comparison, a log-rank test of the effect of chest wall irradiation gave a p-value of 0.06, which is broadly comparable and suggests the Cox model result is adequately robust.

Figure S9: Schoenfeld residuals plot for the randomised treatment parameter in the Cox model for the regional recurrence endpoint

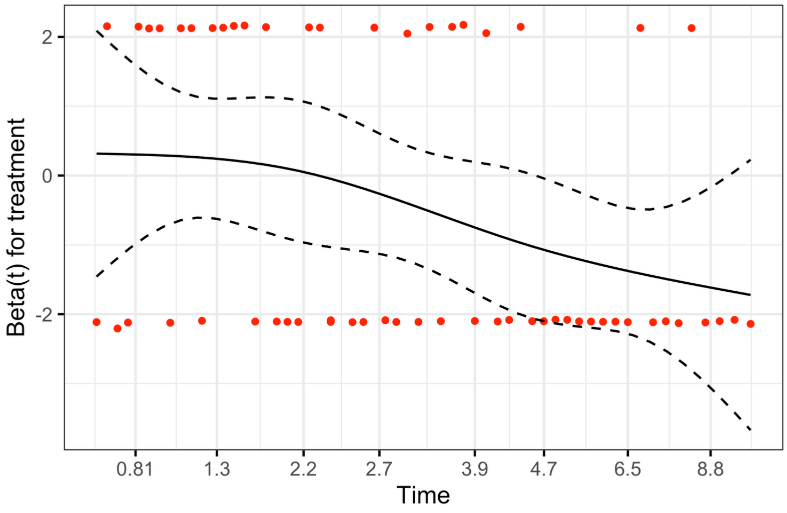

Table S5: P-values for score tests of the proportional hazards assumptions for the three subgroup analysis model sets

Subgroup/ Analysis endpoint	Score test p-value		
	**Randomised Treatment**	**Centre**	**Subgroup**
**Age group**			
Overall survival	0.050	0.087	0.527
Chest wall recurrence	0.111	0.251	0.990
Regional recurrence	***0.011***	0.735	0.944
Metastasis-free survival	0.380	0.314	0.114
Disease-free survival	0.804	0.647	***0.011***
**Nodal status**			
Overall survival	0.056	0.068	0.332
Chest wall recurrence	0.108	0.250	0.511
Regional recurrence	***0.011***	0.732	0.127
Metastasis-free survival	0.408	0.243	0.681
Disease-free survival	0.836	0.538	0.802
**Molecular subtype**			
Overall survival	0.059	0.059	***<0.001***
Chest wall recurrence	0.186	0.242	0.108
Regional recurrence	***0.033***	0.608	***0.007***
Metastasis-free survival	0.402	0.122	***<0.001***
Disease-free survival	0.864	0.422	***<0.001***

For all three subgroup analyses, Table S5 flags the randomised treatment variable for the regional recurrence models – see comments above. The corresponding Schoenfeld residuals plots look very close to Figure S9. It should be noted that these HRs for randomised treatment therefore represent an average over study time of an effect that is changing with study time. The treatment effect does not appear to change direction – it seems to vary between approximately 1 and values lower than 1.

For the age group analyses, the subgroup variable was also flagged in the disease-free survival model as showing evidence of loss of proportionality. A log-rank p-value of p<0.001 was obtained for a comparison of the eight treatment/ age group strata, suggesting this was not an artefact of non-proportionality, and the Schoenfeld residuals plot (shown below in Figure S10A) shows a reasonably horizontal smooth-spline fit, suggesting that this results from outliers rather than a strong non-proportional effect.

For the molecular subtype analyses, the subgroup variable showed strong evidence of loss of proportionality in the overall survival model. A log-rank p-value of p<0.001 was obtained for a comparison of the eight treatment/ subtype strata, suggesting this was not an artefact of non-proportionality. The Schoenfeld residuals plot (shown below in Figure S10B) suggests that the increased hazard (driven primarily by the triple negative subtype) may be relevant only up to 5 years. A similar effect was seen in the regional recurrence model (see Figure S10C; log-rank p=0.005), the metastasis-free survival model (see Figure S10D; log-rank p=0.005) and the disease-free survival model (See Figure S10E; log-rank p<0.001).

Figure S10: Schoenfeld residuals plot for the variables identified in Table S5.
A: Age group for disease-free survival. B: Molecular subtype for overall survival.
C: Molecular subtype for regional recurrence. D: Molecular subtype for metastasis-free survival.
E: Molecular subtype for disease-free survival

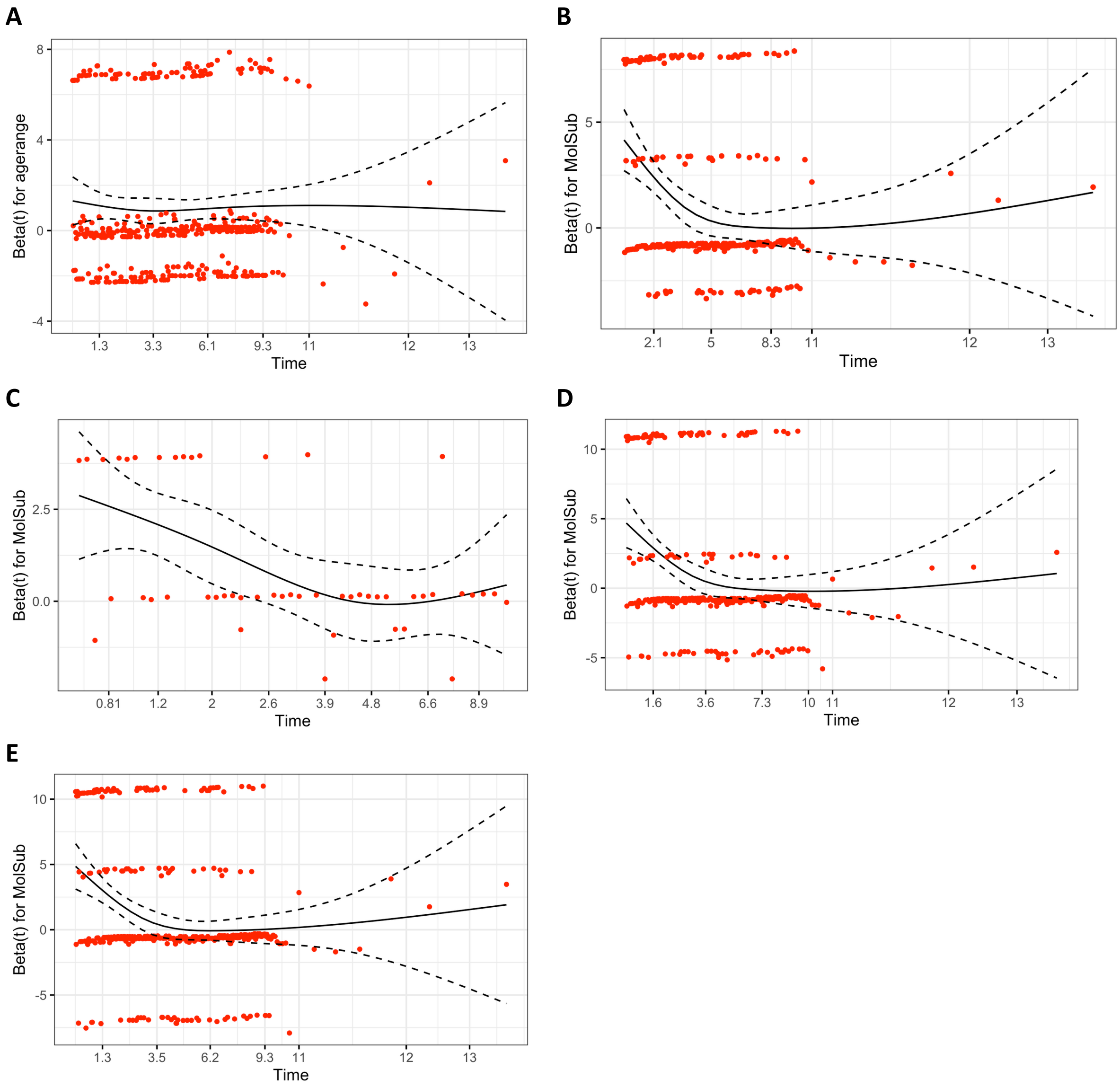

4.4 Assumption of non-informative censoring

It should be noted that the 10-year follow-up period resulted in a considerable quantity of censoring prior to the last visit scheduled for patients at that time point (as distinct from those censored because they were event-free at end of follow-up). This does serve to reduce precision overall, although the impact of this is incorporated in the HR estimates calculated from the Cox models.

The analyses presented in this paper rely on a second important assumption: that the censoring process is not informative. Following the definition in Cook & DeMets (2008)[11], informative censoring would occur when loss to follow-up/ trial discontinuation is dependent on either outcome or treatment. This may be a more plausible situation in a trial such as SUPREMO, where the long follow-up period may permit mechanisms of dependence to operate. Against this, patients were followed up for as long as they were available for us to do so and there was no reason to suppose that patients in the two arms would be followed-up differently in the clinic. Although some smaller scale differences in clinical practice and standards between recruiting sites might have been possible, there was a degree of uniformity of practice across these. The administration (or not) of CWI was a relatively small component of the multi-disciplinary management of the breast cancer patients in the trial and they would have been attending follow-up anyway irrespective of CWI allocation. In addition, as can be seen from the Kaplan-Meier plots in the main paper, the loss to follow-up tended to occur in the latter half of the follow-up period (after 5 years), more removed in time from the administration of randomised treatment. This suggests the censoring was more likely to have resulted from the logistical issues inherent in following patients for 10 years, rather than any factor linked to their randomised treatment allocation. These factors reduce the likelihood of a differential impact on treatment or center effects from this loss to follow-up.

Although it is difficult to demonstrate conclusively that such dependence is absent from trial results, one can also investigate the degree of balance of censoring across treatment arms both graphically and numerically, as well as undertake some straightforward logistic regression modelling to estimate the level of dependence (as suggested by Collett, 1994[12]).

The Kaplan-Meier plots above and in the main manuscript allow a graphical display of censoring rates (vertical tick marks located at each censoring event). Across all endpoints, these appear to be evenly distributed between the arms of the trial, suggesting no gross dependence of censoring time on treatment. Table S6 below displays the overall censoring rates for the endpoints, which suggests the same conclusion.

[11] Cook, T.D. and DeMets, D.L. (2008) Introduction to Statistical Methods for Clinical Trials. London: Chapman & Hall.
[12] Collett, D. (1994) Modelling Survival Data in Medical Research. London: Chapman & Hall.

Table S6: Rates of censoring versus rates of events for primary and secondary endpoints

**Characteristic**	**No Chest Wall Irradiation** N = 799[1]	**Chest Wall Irradiation** N = 808[1]
**Overall Survival**		
Censor	654 (81.9%)	658 (81.4%)
Death	145 (18.1%)	150 (18.6%)
**Chest Wall recurrence**		
Censor	779 (97.5%)	799 (98.9%)
CWR	20 (2.5%)	9 (1.1%)
**Locoregional recurrence**		
Censor	763 (95.5%)	786 (97.3%)
LRR	36 (4.5%)	22 (2.7%)
**DMFS**		
Censor	633 (79.2%)	632 (78.2%)
Metastatic event	166 (20.8%)	176 (21.8%)
**DFS**		
Censor	603 (75.5%)	616 (76.2%)
Disease event	196 (24.5%)	192 (23.8%)

[1]n (%)

Table S7 tabulates censoring rates by the stratification factor for the primary and secondary analyses (regional centers). There is some variation in the rate across center for overall survival, DFS and DMFS, the three endpoints defined in terms of (wholly or in part) mortality. However, the influence of any informativeness here is very low, as unstratified models show the same results in qualititative terms as the stratified ones.

Table S7: Rates of censoring versus rates of events for the centers

**Characteristic**	**British** N = 1,168[1]	**EORTC** N = 323[1]	**International** N = 116[1]
**Overall Survival**			
Censor	942 (80.7%)	269 (83.3%)	101 (87.1%)
Dead	226 (19.3%)	54 (16.7%)	15 (12.9%)
**Chest Wall recurrence**			
Censor	1,146 (98.1%)	317 (98.1%)	115 (99.1%)
CW	22 (1.9%)	6 (1.9%)	1 (0.9%)
**Locoregional recurrence**			
Censor	1,126 (96.4%)	310 (96.0%)	113 (97.4%)
RR	42 (3.6%)	13 (4.0%)	3 (2.6%)
**DMFS**			
Censor	908 (77.7%)	259 (80.2%)	98 (84.5%)
MFSevent	260 (22.3%)	64 (19.8%)	18 (15.5%)
**DFS**			
Censor	875 (74.9%)	249 (77.1%)	95 (81.9%)
DFSevent	293 (25.1%)	74 (22.9%)	21 (18.1%)

[1]n (%)

An additional investigation recommended by some authors is to model the probability of censoring in terms of key explanatory variables using logistic regression models. Table S8 shows the results of this exercise for the primary and secondary endpoints of the trial.

Table S8: Logistic regression models for the probability of censoring for each endpoint, modelled in terms of randomised treatment and center. Model parameters presented as odds ratios, with associated 95% confidence intervals.

**Characteristic**	**OS**			**Chest Wall Rec.**			**Locoregional Rec.**			**DMFS**			**DFS**		
	**OR**[1]	**95% CI**[1]	**p-value**	**OR**[1]	**95% CI**[1]	**p-value**	**OR**[1]	**95% CI**[1]	**p-value**	**OR**[1]	**95% CI**[1]	**p-value**	**OR**[1]	**95% CI**[1]	**p-value**
**Treatment**															
No Chest Wall Irradiation	—	—		—	—		—	—		—	—		—	—	
Chest Wall Irradiation	0.97	0.75, 1.25	0.8	2.27	1.06, 5.28	0.043	1.68	0.99, 2.93	0.058	0.94	0.74, 1.19	0.6	1.04	0.83, 1.31	0.7
**Center**															
British	—	—		—	—		—	—		—	—		—	—	
EORTC	1.20	0.87, 1.67	0.3	1.00	0.43, 2.75	>0.9	0.88	0.48, 1.73	0.7	1.16	0.86, 1.59	0.3	1.13	0.85, 1.51	0.4
International	1.62	0.95, 2.94	0.093	2.16	0.45, 39.0	0.5	1.39	0.49, 5.79	0.6	1.56	0.95, 2.71	0.094	1.51	0.94, 2.53	0.10

[1]OR = Odds Ratio, CI = Confidence Interval

For overall survival, DMFS and DFS, the respective models show no evidence of association between censoring and treatment arm or center. Chest wall and locoregional recurrence have p-values for

treatment close to 0.05. This is interesting, as these two endpoints regard mortality as censoring events. Cook and DeMets (2008) comment that this situation carries more risk of informative censoring, as it may permit a complex interaction between treatment, mortality and outcome. However, these endpoints have low event rates/ high censoring rates (98% & 96% respectively), so have poorer precision and would be sensitive to small numerical changes in event rates. Along with the multiplicity effect introduced by these additional models, they do not provide convincing evidence of informativeness.

This investigation suggests that censoring can be assumed to be non-informative for the SUPREMO trial analyses.
